# Burden on hydropower units for short-term balancing of renewable power systems

**DOI:** 10.1038/s41467-018-05060-4

**Published:** 2018-07-06

**Authors:** Weijia Yang, Per Norrlund, Linn Saarinen, Adam Witt, Brennan Smith, Jiandong Yang, Urban Lundin

**Affiliations:** 1 The State Key Laboratory of Water Resources and Hydropower Engineering Science, Wuhan University, Wuhan, 430072 China; 2 Division of Electricity, Department of Engineering Sciences, Uppsala University, SE-751 21 Uppsala, Sweden; 3 Oak Ridge National Laboratory, Environmental Sciences Division, Oak Ridge, Tennessee USA; 4 Vattenfall R&D, SE-814 26 Älvkarleby, Sweden; 5 Present Address: The State Key Laboratory of Water Resources and Hydropower Engineering Science, Wuhan University, Wuhan, 430072 China

## Abstract

There is a general need to change hydropower operational regimes to balance the growing contribution of variable renewable energy sources in power systems. Quantifying the burden on generation equipment is increasingly uncertain and difficult. Here, we propose a framework combining technical and economic indicators to analyze primary frequency control (PFC) on a timescale of seconds. We develop a model integrating hydraulic, mechanical, and electrical subsystems to characterize efficiency loss, wear and fatigue, regulation mileage, and frequency quality. We evaluate burden relief strategies under three idealized remuneration schemes for PFC, inspired by those used in Sweden, the USA, and China, respectively. We show how burden and compensation vary under future scenarios of renewable power systems. Our framework can be used by producers to develop favorable operation strategies that reduce burden and increase economic value, and by transmission system operators to provide insights on the relation between incentive structures and regulating performance.

## Abstract (web summary)

Quantifying burden on hydropower units for balancing variable renewable energy sources has been uncertain and difficult. Herein Yang et al. propose a framework and characterize the burden, performance and compensation of hydropower regulation of renewable power systems.

## Introduction

With current trends toward de-carbonization in the electricity sector^1^, the growing role of carbon free variable renewable energy (VRE) sources is profoundly changing power systems worldwide^2–4^. Effectively and efficiently dealing with the generation intermittency of VRE is a growing research field^5–8^. Consequences from high VRE integration^9^ will vary between power systems, and can be anticipated on a wide range of timescales. Fewer heavy, synchronously connected generators imply less inertia^10^, crucial for system stability on timescales of seconds. Coupled with atmospheric volatility that produces minute scale variations in VRE output, higher grid frequency volatility occurs on timescales where grid balancing from primary frequency control (PFC) is supposed to be most active—from seconds to minutes. This is the main focus of this paper. Common timescales adopted in studies on feasibility of renewable power systems is down to five-minutes^11^, we place emphasis on shorter-term system dynamic behaviors based on timescale of seconds.

Hydropower, the largest global renewable energy source, shoulders a large portion of the regulation and balancing duty in many power systems, which may include PFC, automatic generation control (AGC), voltage control, spinning reserve, standing reserve, and black start capability^12, 13^. The cost to hydropower units of providing grid ancillary services^14–18^ is approached here for PFC by considering physical characteristics and their relationship with economical remuneration schemes.

From the aspects of the physical features, we focus on (1) increased wear and fatigue^19, 20^ on hydropower machinery and (2) reduced production when efficiency losses are incurred during off-design operation. Wear and fatigue issue have been investigated regarding the service lifetime of runners^21–23^ and bearings^24, 25^ of Francis turbines and Kaplan turbines^26–29^, including the influence of PFC on wear and tear^30, 31^ and strategies on hydro life extension^32^. These works address the issue on detailed components from the aspects of hydraulics and tribology. In terms of reducing efficiency loss, investigations have been conducted based on small to large spatial scale studies, e.g., turbine design^32, 33^ and strategies of operation and power dispatch^34–39^.

From an economic perspective, ancillary service compensation mechanisms for frequency and voltage control are generally provided in regions with mature markets, including Europe (including Sweden), North America (including the USA), Oceania, and South America^12, 40, 41^. PFC is a differentiated product in various countries, generally traded through annual bilateral contracts and tendering processes^12, 40^. Though procurement methods, remuneration methods, remuneration structures, and detailed characteristics of the markets for PFC are categorized and compared^11, 42, 43^, the costs associated with PFC are often difficult to identify, as few regions in the world have an explicit ancillary service market to procure PFC service^42^, and new market designs for PFC service are being actively explored^42–44^. China's electricity market has been developing in recent years^45^ and the importance of ancillary services to VRE integration has become a key issue^13^. However, the Chinese market is still in the exploratory phase^13, 45^, and generally speaking, there is no standard market mechanism for procurement and compensation of ancillary services^13, 46^.

Many previous works highlight the importance of flexible hydropower regulation in achieving high VRE penetration^11, 47, 48^, trends in hydropower operations in the face of growing VRE^49^, and underestimated challenges are discussed^11^; however detailed research to support flexibility assumptions from the control, operation, and economic perspective of the hydropower community itself is rare. In one example, a fully renewable Nordic power system was presumed feasible, from a variability point of view, if hydropower provides proper regulation^9^.

Many hydropower owners are experiencing accelerated degradation of powertrain components due to evolving power system demands for flexibility^50^. A higher volume of balancing actions is driving producers to supervise costs more carefully, and transmission system operators (TSOs) to closely monitor ancillary service quality. As conventional producers operating at cost face lower utilization rates and even negative prices^51^, both producers and TSOs may be motivated to optimize balancing operations for higher revenues. However, the tools and models needed to minimize regulation burden for producers, maintain good regulation performance for TSOs, and achieve reasonable compensation structures for both sides are currently lacking. To bridge this gap, we conduct a systematic study to quantify and evaluate the unforeseen costs and trade-offs between burden and performance of regulation.

Here, we propose a framework combining technical operation strategies with economic indicators to obtain relative values of regulation burden and performance of PFC of hydropower units. To quantify, we establish a numerical hydropower plant (HPP) model with a Kaplan turbine, calibrated with measurements from two Swedish plants (Methods). We study Kaplan turbines since they are more complicated in terms of control (Supplementary Note 1). Hence, the methodology and results can easily be simplified and extended to other turbine types. Comparing to previous relevant works on Kaplan turbines, e.g., efficiency improvement through draft tube design^52, 53^ and control methods optimizations^54, 55^, and operating performance enhancements^56^, our model is interdisciplinary, including dynamics of hydraulic–mechanical–electrical subsystems. We also consider burden relief strategies and their implications under three idealized remuneration schemes for PFC, inspired by those used in Sweden, in parts of the USA, and in East China. They differ in underlying pricing philosophies based on three aspects for PFC: reserved capacity, actual utilization, and a comparison between actual and ideal contribution. Finally, we utilize the model to analyze how regulation burden and compensation for hydropower units could vary under future VRE scenarios.

Our work can be beneficial to both hydropower producers and TSOs: our framework can be a robust tool for producers to achieve favorable operation strategies for relieving burden and obtaining compensation, and we provide insights for TSOs interested in evaluating if their current remuneration structures give the desired incentives. Our framework contemplates power system level and component level issues, covering a wide range of scales under different VRE integration scenarios and operational conditions for different compensation structures from three continents.

## Results

### Framework for quantifying and evaluating the regulation

**Fig. 1:**
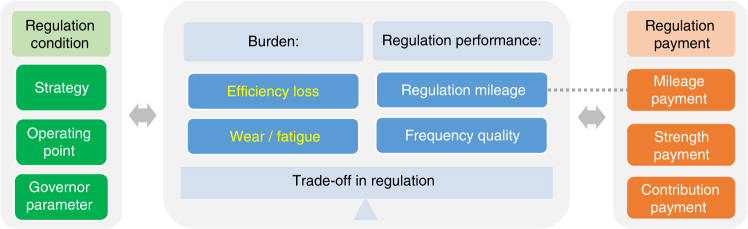
Framework for quantifying and evaluating the regulation of hydropower units. Efficiency loss as well as wear and fatigue are adopted to represent the burden; regulation mileage and frequency quality are applied to evaluate the regulation performance

In the framework (Fig. 1), burden is represented by efficiency loss and wear/fatigue, and regulation performance is evaluated using regulation mileage and frequency quality. The technique to quantify burden and regulation performance and the corresponding indicators that serve as the main outputs of the numerical simulations are introduced in Methods.

Optimizing the regulation conditions of hydropower units is the key to easing their incurred burden. We comprehensively compare various regulation conditions by varying the turbine governor parameters (Ep1 to Ep3 in Supplementary Table 2), operating set-points (seven points in Supplementary Fig. 16) and regulation strategies (Methods). Under different conditions, we concisely analyze the following two idealized pricing schemes of regulation payments: strength payment, mileage payment, and contribution payment that are inspired, respectively, by the ones used by the TSO Svenska Kraftnät (SvK) in Sweden, by PJM Interconnection LLC (PJM) that is a regional transmission organization in the USA^57^, and by East China Energy Regulatory Bureau of National Energy Administration of China^58^. The schemes are detailed in Methods.

**Fig. 2:**
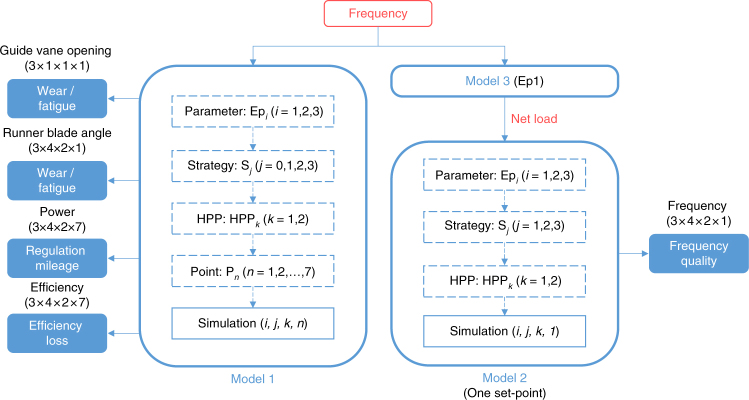
Illustration of the simulation structure. The models 1–3 are introduced in Methods and Supplementary Note 3. The blocks with dashed outline represent the selections in simulation setting based on different conditions: *i* varies from 1–3, for three sets of governor parameters Ep1–Ep3; *j* varies from 1 to 4, for three regulation strategies (S1–S3) and an ideal on-cam case (S0); *k* varies from 1 to 2 for two HPPs; *n* varies from 1 to 7 for seven operating set-points. The set in the parenthesis with simulation presents different cases conducted in the model. In total, there are 168 (3 × 4 × 2 × 7) and 24 (3 × 4 × 2 × 1) simulation cases conducted in Model 1 and Model 2, respectively. The products in the parenthesis with the output variables show the actual needed set of results for analyzing the corresponding indicator in this paper

We adopt the proposed numerical model to conduct various simulations (Fig. 2. and Supplementary Note 6), of which the length is 24 h and timestep is 0.02 s, to test the above-mentioned indicators based on two real HPPs in Sweden (Methods). The input signal of Model 1 and Model 3 is a sequence of measured one-day (24-hour) Nordic grid frequency, and its sampling time is 1.0 s. The net load^9, 59^ and the frequency are introduced in Supplementary Note 3 and Supplementary Note 6 (including Supplementary Fig. 14 and Supplementary Fig. 15). All the variables in this paper are introduced in Nomenclature in Supplementary Note 1.

### Burden quantification

In this section, we discuss the efficiency loss, as well as wear and fatigue due to regulation. The overall results of this study under various operation conditions are shown in Table 1, and detailed results of efficiency loss are presented in Table 2. It is shown that the governor setting directly influences the efficiency loss: the lower the droop (the higher the static gain), the more efficiency loss. However, the loss is not linearly related to the droop due to the complexity and nonlinearity in the system.

**Table 1:** Overall results of different operation conditions from one-day simulation

The bar in each cell indicates the relative magnitude of the values with the same color. The results of efficiency loss are condensed from Table 2.The results of guide vane (GV) movement are based on HPP 1, because there is little difference on the indicators between two HPPs. The operating point does not influence movement of GV and runner blade (RB) much, hence only the results from point 5 are shown. For frequency quality, the values of the change of root mean square error are shown, and they are condensed from the detailed values in Supplementary Table 5; negative and positive values are shown with green and gray bars, respectively, and positive values indicate better frequency quality. The regulation mileages are shown with purple bar, and the detailed results are in Fig. 4. The “pu” is short for per unit through out of this paper.

**Table 2:** Detailed simulation results of efficiency change (Δ*η*_Sj_)

The seven operating points of each HPP are shown in Supplementary Fig. 16. Negative values mean efficiency loss. The bar in each cell is a graphical representation of the value, and the negative and positive values are highlighted by red and blue, respectively. The average values of seven points are selected as the representative results listed in Table 1. The value of “Max–Min” is the difference between the maximum and minimum of the efficiency changes of seven points.

We investigate the composition of efficiency loss in regulation by comparing results across different strategies. For the normal PFC (S1) in HPP 2, the maximum efficiency loss due to regulation reaches 0.44%, 0.63%, and 1.3% under Ep1, Ep2, and Ep3 setting, respectively (0.27%, 0.49%, and 0.10% for HPP 1). The difference between efficiency losses under S1 and S0 (strict on-cam) is very small, indicating that the loss due to normal PFC is mainly caused by the trajectory deviation from the set-point. The extra loss due to off-cam operation is negligible for normal PFC, but not for wear reduction strategies. The operation strategy S3 (no RB movement) leads to a considerable efficiency loss that is larger than 1.9% under the high gain setting Ep3 and mainly caused by off-cam operation, showing the economic drawback of the strategy. While the strategy S2 (small floating dead-zone on runner control) only causes a slight increase (~0.03 %) of efficiency loss compared to S1. It should be noted that relatively minor changes in efficiency could have significant fleet-wide impacts, due to the huge scale of hydropower capacity in many power systems (Supplementary Note 9).

**Fig. 3:**
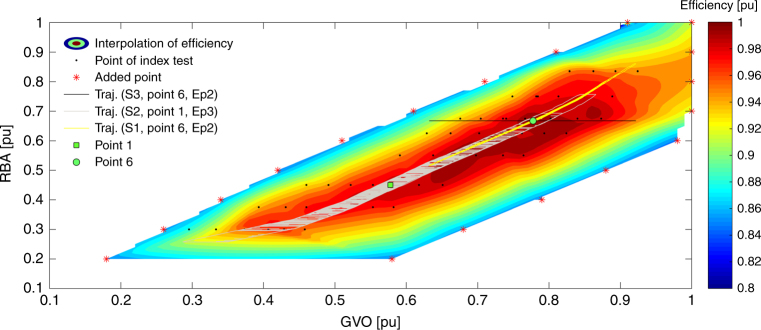
Efficiency contour of HPP 2 from interpolation of the efficiency measurements and the operation trajectories during the 24 h. The trajectories are from operation strategies S1–S3 with different operating points and governor parameters (Ep2 and Ep3). The efficiency value is normalized with respect to the maximum efficiency value. The black dots show the locations of the operating points in the index test, corresponding to the blue markers in Supplementary Fig. 7 and Supplementary Fig. 8

More specifically, the turbine efficiency and the trajectory of the GV opening (GVO) and RB angle (RBA) under three simulated operating strategies are shown in Fig. 3. The yellow trajectory describes the normal PFC (S1) with the set-point selected as point 6. In this case, the off-cam phenomenon is caused by the mechanical system, e.g., backlash, delay, and servo. A more obvious off-cam operation is presented by the gray trajectory, from the S2 with a filter for RBA (0.03 pu floating dead-zone). The black trajectory shows the extreme strategy S3 that is fixing the RBs, and the trajectory is out of the measurement range, demonstrating the need of extrapolation from the fitting of efficiency.

We found that the operating set-point has a considerable influence not only on the steady-state efficiency but also on the efficiency loss in transients, as shown in Table 2. The differences between the maximum and minimum of efficiency changes under seven points are considerable (e.g., 0.800%, 1.112%, and 1.637% under Ep1 through Ep3, respectively, for HPP 2). This causes complexity in the quantifying and evaluating process. In some cases, the efficiency even increases compared to the steady-state value, as highlighted by blue in the table cell. The main explanation is that the on-cam efficiency of a set-point (e.g., point 1) is not the global maximum. During the operation, the trajectory enters into the region with higher efficiency, for example, the right upper part of the blue trajectory in Fig. 3. Supplementary Fig. 18 presents this case in a clearer manner: the instantaneous efficiency can be higher than the steady-state value (see blue lines for point 1). It is also indicated that the set-point may not be the local maximum, due to a non-optimal combinator, leading to a more obvious efficiency increase. While for point 6 where the on-cam operation has a high efficiency (close to the global maximum), the instantaneous efficiency is mostly lower, and the efficiency loss is larger than 0.4% under a very favorable operation case, namely strategy S1 with parameter set Ep1. It is worth emphasizing that a non-optimal combinator would result in less efficiency loss. Meanwhile, performance improvement projects^32, 56^ are common nowadays for obtaining a better combinator to achieve higher on-cam efficiency, hence the off-cam efficiency loss would be even larger due to these works.

Since the magnitude of the efficiency loss is small, it is necessary to discuss uncertainties. There are two main potential error sources. First, the error of efficiency measurement in the index test is stated to be 0.2%, which is close to the highest accuracy that can be achieved in the current hydro turbine field. Second, extrapolation from data fitting inevitably causes errors. However, the influence of these errors in individual data points is counteracted and decreased in the simulation cases of which the operation trajectory covers multiple operating points during one day. Moreover, the influence of the water head and turbine rotational speed on the turbine characteristic is ignored due to the limitation of on-site measurement data, but it is acceptable for the small disturbance conditions of this study.

In terms of wear and fatigue, the governor setting has a direct influence, as shown in Table 1. A higher droop leads to longer distance and larger amount of movements. For the GV movements, results do not differ from S1 to S3, because different strategies only affect the RB side. The burden relief strategy S2 leads to a significant decrease of both the distance and amount of RB movements, and strategy S3 totally diminishes the RB movement. The effect of strategy S2 is demonstrated in time-domain in Supplementary Fig. 19. Besides, the influence from different operating points is small, and this decreases the complexity in analysis. On this stage, we only investigate the total distance and amount of movements to reflect the wear and fatigue; the simplified burden here can be extended by studying more influencing factors, e.g., cavitational operation, different wear types, etc. The correlation between these indicators to the actual damage, maintenance time, and lifetime estimation of components is an important future work.

### Regulation performance

**Fig. 4:**
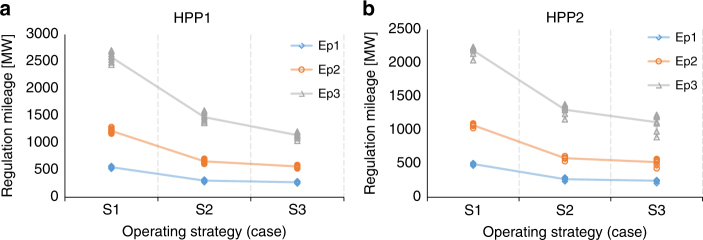
Regulation mileage of two HPPs for 24 h. For each case, results from all seven operating points are close, hence the scatters overlap each other; the average values of seven points in various conditions are presented in Table 1. **a** For HPP 1; **b** For HPP 2

In this section, we discuss the regulation mileage and frequency quality as crucial trade-off perspectives of the burden. As shown in Table 1 and Fig. 4, there is a high correlation between regulation mileage and static gain, 1/*b*_*p*_ (Methods). As to the operating strategy, the wear reduction strategy S2 and S3, especially S3, lead to decreases in mileage. The results are not influenced much by the operating set-points, and this is conducive to simplify the evaluation. In terms of frequency quality, the results are presented in Table 1 and Supplementary Table 5. A larger value indicates a better frequency quality. Overall, lower droop that corresponds to higher regulation strength of the unit results in better frequency quality, based on our setting that the regulation strength of the remaining units in the grid is unchanged. The influence of off-cam operation is shown clearly: when S2 is applied, the frequency quality worsens compared to the performance of S1 under the same Ep setting. The deterioration of the frequency quality is more obvious under S3.

Comparing the frequency quality and mileage between the two cases S1 with Ep1 and S2 with Ep2, one can observe that the latter case leads to a much better frequency quality (+0.45 % for HPP 1) with a slightly larger mileage. It demonstrates an advantage of adopting S2. This also implies that the mileage mainly represents the quantity, instead of quality, of the regulation and both the mileage and frequency quality are necessary for ensuring preciseness of evaluation.

### Regulation payment

Evaluating consequences of regulation payment schemes is of great importance for both power producers and TSOs. Here, we analyze three idealized schemes (Methods) inspired by the payment schemes of SvK in Sweden, PJM in the USA, and the National Energy Administration in China based on their relative payment values. Relative values of payments under different conditions are presented in Table 1, and detailed results of regulation strength and contribution payment are presented in Supplementary Table 6 and Supplementary Table 7, respectively.

Both strength payment and mileage payment lead to compensation overall aligned with the performance, as it correlates well with RB movement and frequency quality. Contribution payment reflects the performance well, when applying different regulation strategies; nevertheless, it has an opposite trend with the performance under three settings of governor parameters, because the pricing philosophy is based on the comparison with the ideal value that also varies with governor settings, instead of focusing on indicators with absolute values as in the other two payment schemes.

Compared to strategy S1, strategy S3 leads to a considerable compensation decrease while causing an obvious drop in efficiency and poor frequency quality. Therefore, S3 may not be a promising choice for producers. Alternatively, strategy S2 can be suitable for producers, because it does not result in significant compensation decline (especially for HPP 2) and can largely decrease the RB movements without deteriorating the efficiency and frequency quality too much. An important finding is a large difference in different payments under burden relief strategies S2 and S3. This illustrates the influence that payment schemes might have on operating strategies chosen by producers.

**Fig. 5:**
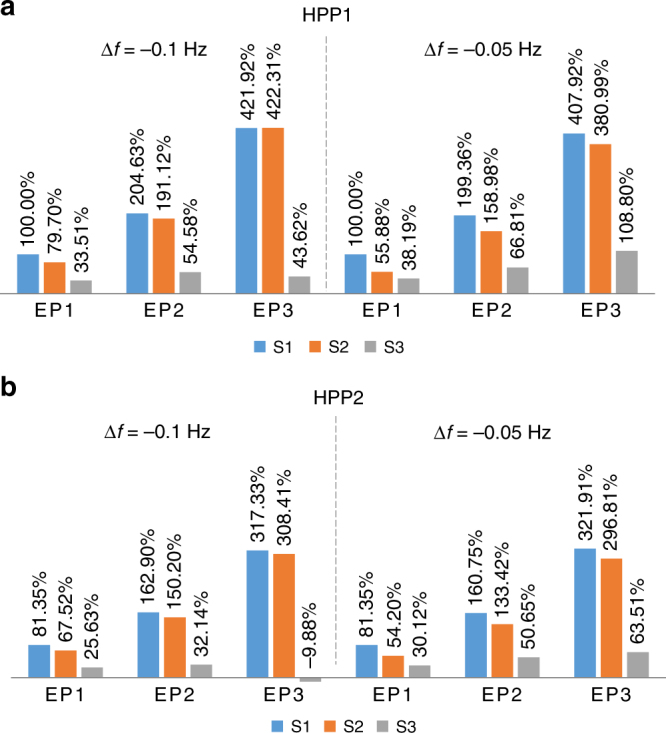
Strength payments based on the strength obtained from the −0.1 Hz frequency step change and a −0.05 Hz frequency step change. For each frequency change case, the relative values are normalized with the respect to the strength under Ep1 and S1 for HPP 1. **a** For HPP 1; **b** For HPP 2

In terms of strength payment, the value for HPP 2 under S3 and Ep3 is negative, because the turbine efficiency sharply decreases in the off-cam operation and the power output after the frequency change is even smaller than the original value. However, the regulation performance of the case is positive (with +0.38% in frequency quality and 958.6 MW in mileage) since the average frequency deviation is considerably smaller than the 0.1 Hz used to determine strength. This demonstrates a limitation of strength payment: it ignores the performance of actual delivered regulation. As shown in Fig. 5, the negative strength is avoided by varying the frequency step change to −0.05 Hz from the original value 0.1 Hz: the payment increases to 63.5% from −9.9% under S3 with Ep3 for HPP2. This reflects the complexity of achieving an appropriate implementation of strength payment.

Intricate results are found for contribution payments as well. Both the ratio of contributed regulating energy (*λ*_R-avg_) and the score of regulation correctness (*λ*_C_) decrease from Ep1 to Ep3 (Supplementary Table 7), showing that the difficulty to achieve high scores increases with the rise of the static gain; a main reason is that the amount of the effective regulation movements (Supplementary Note 8) increases. Besides, the values of *λ*_C_ under strategy S3 are generally larger than the ones under strategy S2; under Ep3, the payments for strategy S2 are even lower than the ones for strategy S3. A core cause for the complex outputs is that the pricing philosophy are more sensitive to physical features in the hydropower systems.

**Fig. 6:**
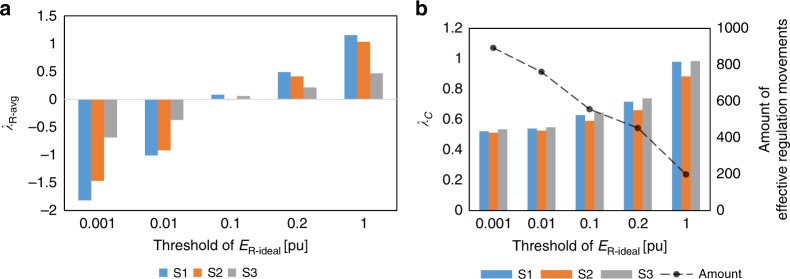
Results regarding contribution payment under different thresholds of *E*_R-ideal_ (for HPP 1 under Ep2): **a** average ratio of contributed regulating energy (*λ*_R-avg_) (**b**) ratio of regulation correctness (*λ*_C_) and the amount of effective regulation movements. Detailed values of *λ*_R-avg_ and *λ*_C_ are shown in Supplementary Table 7

Another key factor in contribution payment is the threshold of *E*_R-ideal_ for defining the effective regulation movement (Method and Supplementary Note 8). As shown in Fig. 6, the increase of the threshold reduces the amount of effective regulation movements and improves both indicators, especially the main indicator *λ*_R-avg_, which varies significantly from negative to positive values, reflecting the necessity of applying the threshold. This demonstrates that the payment value is strongly influenced by detailed settings of the remuneration scheme, revealing a benefit of applying our model as a tool for optimization.

### Future scenarios of VRE integration

High VRE integration leads to generation intermittency, less inertia, and affects the damping of electro-mechanical oscillations in power systems. These three aspects contribute to grid frequency deviation, which is a representative indicator of collective dynamics and stability of power systems^60^. For the Nordic power grid, the number of minutes per week with the grid frequency outside the normal bandwidth has a clear growth trend since 2001, demonstrating that maintaining the frequency level at 50 ± 0.1 Hz at all times is increasingly difficult for system operators^4^ (Supplementary Fig. 21); Meanwhile, the increase of VRE production portion from 2001 to 2013 is shown in Supplementary Fig. 22, and it is one of the causes of worsening frequency quality.

First, regarding the increased generation intermittency and net load variability (Supplementary Note 3) caused by the enlarged share of VRE^9, 59^ in power systems, much previous research focused on the characteristics^9, 61, 62^ and countermeasures from hydropower^9, 63, 64^ based on long timescales, and very few studies addressed sub-hourly scales^61, 62^. Standard deviations of the net load of the Nordic power system in various VRE scenarios based on different timescales (>daily) are quantified, and the value could be two times larger than the current case^9^; VRE variability and nonlinearity in the timescale of seconds were investigated^65^, however the explicit relationship between the VRE integration and net load variability in the sub-hourly scale is still unclear. Second, under future VRE integration scenarios of the Nordic power system, the Nordic TSOs estimate that the kinetic energy (which indicates system inertia) of the system during low loads (typically summer nights) will be 124;GWs in 2020 and possibly as low as 80 GWs in 2025. This is much lower than the 250 GWs of the current system^10^. Such a significant reduction in system inertia will go against stability of power systems and demand more regulating work. Third, electro-mechanical oscillations in power systems from wind and PV penetration can lead to both detrimental and beneficial impacts^66, 67^; burden on hydropower units from negative damping contribution of VRE integration should be concerned and analysed.

**Fig. 7:**
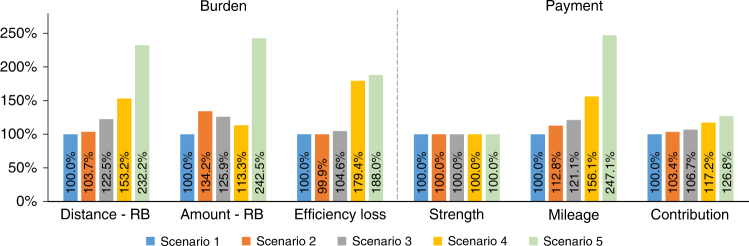
Relative values of the burden and the payments under different scenarios. The results for HPP 1 under Ep1 and S1 are selected and shown. The distance and the amount refer to the corresponding values for RB movements

In this section, we apply deteriorated frequency sequences (mainly caused by the net load variability, the inertial, and the damping) as simulation model inputs (Supplementary Note 10 including Supplementary Table 8 and Supplementary Fig. 23) to study the influence of future VRE scenarios on regulation burden and compensation for individual hydropower units in the timescale of seconds. Relative values of the burden and the payments under different scenarios are shown in Fig. 7, and detailed results are presented in Supplementary Table 9 and Supplementary Table 10, respectively.

In terms of the burden, wear and fatigue aspect indicated by the distance and the amount of GV and RB movements are significantly aggravated under the future scenarios. More exactly, the amount of RB movements under Scenario 5 (with the worst frequency quality) and strategy S1 is more than twice of the value under Scenario 1 for the current condition; while this can be well resolved by applying strategy S2 (Supplementary Table 9). Meanwhile, Scenario 4–5 show that the increased net load standard deviation leads to more efficiency loss, however the influence from the inertia and the damping of the system on the efficiency loss is less than 5%, because the frequency sequences of Scenarios 2–3 mainly increase the denseness of the turbine operation trajectory rather than expand it the into low efficiency areas.

As to the payment, first, strength payment inherently does not change under different frequency sequences, revealing a key drawback of it. Second, mileage payment well captures the growth trend of the burden due to its good agreement with the movements of turbine actuators. Third, contribution payment to some extent responds to the different scenarios, especially under the high static gain (Ep3) and S1.

## Discussion

Within the proposed framework, we conducted comprehensive quantification of the burden and quality of hydropower regulation through simulations of two Swedish HPPs. Aiming at the system dynamic behaviors in timescale of seconds, we characterized the efficiency loss, wear and fatigue, regulation mileage, and frequency quality with respect to different operating conditions, including governor droop, operating strategy, and operating set-point. We evaluated burden relief strategies under three idealized remuneration schemes for PFC and analyzed regulation burden and compensation under different future VRE scenarios.

A complexity of the quantification lays in the influence of the physical features in the hydropower system, especially the turbine characteristics. The efficiency loss, GV and RB movements, regulation mileage, performance score, and frequency quality differ across operating conditions and turbines, indicating that site-specific analysis may be necessary to quantify the burden of regulation at each HPP. While our refined and interdisciplinary model incorporates precise HPP features, it can flexibly adapt to diverse cases and is easily revised to analyze different turbine types. By developing a framework using Kaplan turbines, machines with high complexity due to double-regulated gates and blades, an extension to more simple and common turbine types, such as Francis turbines, is straight forward. Analysis for other purposes, e.g., analysis on safety and stability, and quantifications of additional variables of interest is feasible as well. Ideally, an on-site experimental investigation could produce more accurate and stricter results by including more operating points to achieve an efficiency distribution over a wider operational range. However, this type of study is difficult to accomplish due to the huge costs of conducting the tests. In short, the methodology adopted in this study effectively makes the most of the current state of the industry.

Our work provides insights into the relationship between regulation conditions and remuneration schemes, benefitting both hydropower producers and TSOs. From the standpoint of producers, our model can be a solid tool to develop favorable operation strategies for relieving burden and procuring compensation. Another benefit is the possibility to evaluate remuneration schemes. An example is that the burden relief strategy S2 can be considered for implementation, due to its favorable overall performance on wear reduction and regulation performance. Decisions along that line will, of course, depend on the economic consequences related to the remuneration method.

For TSOs, remuneration analyses using this framework can be used to formulate detailed operating guidelines or best practices for HPPs. The results can be evaluated to investigate whether the different implementations of different remuneration philosophies give the desired incentives and deliveries. The three payment approaches examined here represent different pricing philosophies: strength payment mainly compensating for reserved capacity by scaling the clearing price with the regulation strength; mileage payment evaluating the actual utilization with respect to regulation mileage; and contribution payment lies on comparisons between actual and ideal regulations, instead of absolute values (the strength or the mileage) as in the other two payment schemes. These philosophies correspond to two remuneration structures, e.g., availability and utilization^40^ or capacity and use^12^. None of the schemes is affected directly by variations in efficiency loss, which varies with turbine characteristics and operating points. It is up to the producers to reflect that in the clearing price. Wear, on the other hand, can be expected to correlate to mileage, and is hence influencing the mileage payment directly, whereas this factor is hidden in the clearing price in strength payment. Correlation between the burden and the remuneration is less regular under contribution payment, due to its high dependency on physical features in the system and detailed settings of the remuneration scheme (e.g., the threshold of the effective regulation movements).

Our work could relatively quickly impact the operation of HPPs in existing power systems with increasing renewables penetration. In the US, the lack of proper incentives for PFC are discussed^42, 44^, and the value of hydropower to the grid is a subject gaining increased attention, where aging HPPs are expected to alter their performance in new and unknown ways^15^. For China's ongoing electricity market reforms, the lack of proper mechanisms for procuring ancillary services for VRE integration is a key issue currently and gets more and more attention^13, 45, 46^. Within the Nordic TSOs, further development of the current markets is necessary with the emerging challenges from growing disconnect between electricity supply and market demand^4, 68^. The value of this manuscript is thus its immediate applicability to entities with both hydro and non-hydro assets.

In this study, we build a bridge between technical operation and economic evaluation, as an initial step. Additional work on economic values would go a long way towards the ultimate goal: holistic quantification of the cost of frequency control. We hope that our work triggers greater attention to and participation in the study of hydropower regulation to produce an effective and efficient operation for balancing renewable power systems.

## Methods

We clarify the scope of this study as follows. The only operational control approach assessed is PFC, and other operation cases (e.g., start-up, stopping, and secondary frequency control) are not considered. The burden (efficiency loss, wear, and fatigue) is investigated from a physical perspective. Further economic modeling to assess the gain or loss of profit from regulation is necessary in the future to fully characterize the effects of PFC on system economics. Only one single unit of a HPP is analyzed in the model, while the model can be conveniently extended to analyze multiple-unit scenario. All the variables in this paper are introduced in Nomenclature in Supplementary Note 2.

### Numerical model

The numerical models built in this study are presented in Supplementary Note 3 (including Supplementary Fig. 1 to Supplementary Fig. 4 and Supplementary Table 1). Model development regarding the turbine characteristics is a key point in this study and is introduced in Supplementary Note 4 (including Supplementary Fig. 5 to Supplementary Fig. 8) based on data from two practical cases presented below. The model validation is conducted based on comparisons of measurements and simulations, as shown in Supplementary Note 5 (including Supplementary Fig. 9 to Supplementary Fig. 13). It should be noted that in this work, the floating dead-zone is applied to the runner control only; frequency dead-zone (dead-band) is not included according to the case in the Nordic power grid, while it is utilized in many other countries including the USA^41^ and China.

### Practical cases: two HPPs

We use two Swedish HPPs (HPP 1 and HPP 2), owned by Vattenfall, the largest hydropower owner and operator in Sweden, as the engineering cases in this study. HPP 1 contains two generating units with Kaplan turbines, while HPP 2 has three units with Kaplan turbines. One unit of each HPP is taken as the study case. The basic information and parameter values of the two HPPs are shown in Supplementary Table 3 and Supplementary Table 4. The combinator table, implemented in the governor of the HPPs as a lookup table for determining the RB angle, is illustrated in Supplementary Fig. 16. Applying more than one engineering case is necessary for this study. The main reason is that the analysis and result of efficiency loss are highly dependent on the turbine unit characteristics, which vary significantly across different turbines.

### Regulation strategies

We analyze the following operation strategies S1–S3 and an ideal case S0. (1) S1: normal PFC in which GV and RB regulate without any artificial filter (widely implemented); (2) S2: PFC with a floating dead-zone filter (0.03 pu) for reducing the movement of RBs; (3) S3: PFC with the RBs being totally fixed (no RB movements); and (4) S0: Normal PFC in an ideal on-cam condition. Now-a-days, the implementation of burden relief strategy (S2 and S3) is under discussion in industry^27^, to avoid the serious fatigue issue on the RB and the hub. For strategy S2, the performance can be altered by changing the setting of the filter, and in this study we only analyze one representative case of S2; also, the filter is: a floating dead-zone that can lead to overall good regulation performance^31^ but not yet implemented in power plants, instead of normal dead-zone that is widely used in the world, especially in Chinese HPPs. The ideal case S0 is unrealistic and only implemented to identify the off-cam loss in normal PFC. Detailed settings are in Supplementary Note 6.

### Method of quantification

Here, we introduce the method of quantification of the burden and quality of regulation. We quantify various indicators through simulations (Fig. 2) under three governor parameter sets (*i* = 1, 2, 3 for Ep1–Ep3), seven operating points within the maximum efficiency range (*n* = 1, 2,…, 7) and four regulation strategies (*j* = 0, 1, 2, 3 for S0–S3) for two HPPs (*k* = 1, 2 for HPP1 and HPP2). The detailed settings of the corresponding simulations are found in Supplementary Note 6.

We simply present the influence mechanism of the governor parameters and the focus is on the droop (*b*_*p*_). For a PI (proportional-integral) controller with droop, the transfer function is

1 $$\frac{{\Delta y(s)}}{{\Delta f(s)}} = - \frac{{K_{\mathrm{p}}s + K_{\mathrm{i}}}}{{(1 + b_{\mathrm{p}}K_{\mathrm{p}})s + b_{\mathrm{p}}K_{\mathrm{i}}}}$$

Here, Δ*y* and Δ*f* are the GVO deviation and frequency deviation from set-point value, respectively, and *b*_p_, *K*_p_, and *K*_i_ are the governor parameters. The corresponding gain is

2 $$G_{{\mathrm{PI}}} = \frac{1}{{b_{\mathrm{p}}}}\sqrt {\frac{{1 + \left( {\frac{{2\pi }}{{T_{\mathrm{f}}}}} \right)^2 \cdot \left( {\frac{{K_{\mathrm{p}}}}{{K_{\mathrm{i}}}}} \right)^2}}{{1 + \left( {\frac{{2\pi }}{{T_{\mathrm{f}}}}} \right)^2 \cdot \left( {\frac{{1 + b_{\mathrm{p}}K_{\mathrm{p}}}}{{b_{\mathrm{p}}K_{\mathrm{i}}}}} \right)^2}}}$$

which has a highly positive relationship with the GV movement distance^30^ and the regulation mileage. *T*_f_ is the period of the frequency oscillation. Equation (2) also shows that if the period (*T*_f_) is large enough, the gain approaches 1/*b*_p_. The value of 1/*b*_p_ is regarded as the static gain in this study and it significantly affects the relative amount of strength payment.

Efficiency loss: We classify the loss into the following compositions (Supplementary Fig. 17). The loss in steady-state operation, −Δ*η*_st_, is

3 $$- \Delta \eta _{{\mathrm{st}}} = 1 - \eta _{{\mathrm{st}}},\left[ {{\mathrm{pu}}} \right].$$

A negative value of the efficiency change indicates an efficiency loss. The “pu” is short for per unit. The *η*_st_ is the on-cam steady-state efficiency that is a constant value taken from the interpolation, and it varies for different operating points. It is not within the interests of this study because it is not influenced by transients in regulation. Here we mainly analyze the extra efficiency loss due to regulation, which is given as

4 $$- \Delta \eta _{{\mathrm{Sj}}} = \eta _{{\mathrm{st}}} - \eta _{{\mathrm{Sj}}},\left[ {{\mathrm{pu}}} \right].$$

Here, the *η*_Sj_ is the average value of the instantaneous efficiency during the operation period (one day in this study) under a specific strategy (S_j_). More exactly, the efficiency loss in transient due to deviation from the set-point (on-cam) can be obtained as

5 $$- \Delta \eta _{{\mathrm{S0}}} = \eta _{{\mathrm{st}}} - \eta _{{\mathrm{S0}}},\left[ {{\mathrm{pu}}} \right].$$

The loss due to off-cam in normal PFC is achieved by the difference between Δ*η*_S0_ and Δ*η*_S1_. The extra loss due to off-cam condition under strategy S2 and S3 is the difference between Δ*η*_S0_ and Δ*η*_S2_, and the difference between Δ*η*_S0_ and Δ*η*_S3_, respectively.

Wear and fatigue: For quantifying the wear and fatigue of turbines, we use the following two indicators^30, 31^ for both GV and RB. The first is the movement distance that is the accumulated distance of movements of GV and RB^31^, as described in

6 $$\left\{ \begin{array}{l}Y_{{\mathrm{GV,dist}}} = \mathop {\sum}\limits_{{\mathrm{is}} = 1}^N {\left| {y_{{\mathrm{is}}} - y_{{\mathrm{is - 1}}}} \right|} \\ Y_{{\mathrm{RB,dist}}} = \mathop {\sum}\limits_{{\mathrm{is}} = 1}^N {\left| {a_{{\mathrm{is}}} - a_{{\mathrm{is - 1}}}} \right|} \end{array} \right.$$

Here, N is the total amount of samples and is means the sample number. *y* and *a* represent GVO and RBA, respectively. From the standpoint of tribology, there is a linear positive correlation between movement distance and material deterioration on bearings^25^. The second indicator is the movement amount that is the total amount of movements, corresponding to the amount of direction changes. A large amount of actuator movement leads to dynamic loads on the turbine runner^22, 69^, and it also implies a multitude of load cycles that might increase the structure fatigue.

On this stage, applying the two representative and concise indicators of wear and fatigue is beneficial to the applicability of the model and the framework, based on simplifications to some extent. Influence mechanisms of detailed patterns of the regulation movements on lifetime and maintenance period of hydropower units is of great importance for future work.

Regulation mileage: We introduce regulation mileage to quantify the amount of work hydropower units expend to follow a regulation signal. The regulation mileage is described in

7 $$M_{\mathrm{R}} = P_{{\mathrm{m - rated}}}\mathop {\sum}\limits_{is = 1}^N {\left| {p_{{\mathrm{m,is}}} - p_{{\mathrm{m,is - 1}}}} \right|} ,\left[ \mathrm{{MW}} \right].$$

Here, *p*_m_ is the active power in per unit; *P*_m-rated_ is rated power of the Kaplan turbine, and its unit is MW.

Frequency quality: We apply the frequency quality^31^ to comprehensively reflect the regulation performance of the hydropower unit, instead of utilizing the response time^70^ or phase shift of the regulation power. As shown in the lower part of Fig. 2, the core idea is comparing the new frequency sequence of the power system under different regulation conditions, to examine whether the frequency quality is deteriorated (Supplementary Note 6). The frequency quality is evaluated through the mean value, standard deviation, and root mean square error (RMSE with respect to the rated frequency 50 Hz) of the frequency sequence. The RMSE is taken as the main indicator. This method examines the influence of the regulation from a single Kaplan unit on the frequency of the whole grid.

### Regulation payment

In this study, we only consider relative values of payment. Clearing prices are not considered in the quantification. The time of commitment and determination of payments for the cases in different countries are introduced in Supplementary Note 7.

Strength payment *P*_strength_, inspired by SvK, is here computed as

8 $$\left\{ \begin{array}{l}S_R = \frac{{P_{{\mathrm{step}}}}}{{0.1}}{\mathrm{,[MW/Hz]}}\\ P_{{\mathrm{strength}}} = \frac{{S_{\mathrm{R}}}}{{S_{{\mathrm{R - base}}}}},{\mathrm{[pu]}}\end{array} \right.$$

Here *S*_R_ is the regulation strength; *P*_step_ is the increase in output power caused by a frequency step change that is selected as from 50 Hz to 49.9 Hz by SvK. *S*_R-base_ is a base value for normalizing the payment, and here it is set to the regulation strength (41.08 MW/Hz in Supplementary Table 6) of the unit in HPP 1 under Ep1 and S1. The payment will depend on governor parameterization, turbine characteristics, burden relief strategy, and operating point. Note that equation (8) considers the available reserve since *P*_step_ will be affected by operational limits. The real hourly compensation for PFC is determined by this measure multiplied by the clearing prize, which is set on a pay-as-bid market and is supposed to reflect the costs for reserving and delivering the service. A contract between SvK and pre-qualified producers state the factors that can motivate the bids, including efficiency losses, lost income due to non-optimal planning with respect to electricity market prices or spill, and equipment wear. The contract also allows for some profit margin and a risk margin motivated by e.g., failures and prognosis uncertainties. There is also a second part of the real compensation from SvK, intended to adjust the above with respect to the extra energy spent or saved when delivering the service. The money related to the adjustment can hence go either way. This part, even though it depends on the actual operation, is omitted in this study, since its magnitude is considerably smaller than the strength payment part, and since its outcome is mainly determined by the signed mean frequency deviation.

Mileage payment: *P*_mile_, inspired by PJM, is based on the mileage in power output

9 $$P_{{\mathrm{mile}}} = \frac{{M_{\mathrm{R}}}}{{M_{{\mathrm{R - base}}}}},\left[ \mathrm{pu} \right].$$

*M*_R-base_ is a base value for normalizing the payment, and here it is set to the regulation mileage (449.5 MW in Table 1) of the unit in HPP 1 under Ep1 and S1. The actual scheme used by PJM does not measure the output mileage, but rather the mileage of a control signal that is computed from frequency deviation and area control error and distributed by PJM. The actual scheme also scales the mileage by a performance score, depending on comparisons between the control signal and power output, rewarding precision, correlation, and a small delay. Regulation mileage and performance are then multiplied by a market-driven clearing price to set the total regulation compensation.

Contribution payment: *P*_contrib._, inspired by East China Energy Regulatory Bureau of National Energy Administration^58^, is introduced here. The core idea, as shown in Supplementary Fig. 20, is to compensate regulation performance of hydropower units based on a comparison between the actual amount of electrical energy used for PFC and an “ideal” value.

For an arbitrary regulation period, the energy that contributes to regulation is defined as

10 $$E_R = \mathop {\sum}\limits_{{\mathrm{is}} = N_{{\mathrm{start}}}}^{N_{{\mathrm{end}}}} {\left( {p_{{\mathrm{m,is}}} - p_{{\mathrm{m - set}}}} \right)} \times \Delta t$$

Here, *N*_start_ and *N*_end_ indicate the sample number of the start and the end of a period, respectively, and *p*_m-set_ is the set-point of power output, and *p*_m,is_ is the turbine active power at timestep is. The value of *E*_R_ can be demonstrated as the area of the gray region in Supplementary Fig. 20. The ideal value of the energy for regulation, *E*_R-ideal_, is described as

11 $$E_{{\mathrm{R - ideal}}} = \mathop {\sum}\limits_{{\mathrm{is}} = N_{{\mathrm{start}}}}^{N_{{\mathrm{end}}}} {\Delta p_{{\mathrm{m - ideal}}}} \times \Delta t.$$

Here, the ideal power deviation for the regulation Δ*p*_m-ideal_ is defined according to the steady-state characteristics of governor speed droop, as shown below

12 $$\Delta p_{{\mathrm{m - ideal}}} = - \frac{{\Delta f_{{\mathrm{is}}}}}{{b_{\mathrm{p}}}}.$$

As shown on the top axis of Supplementary Fig. 20, the whole evaluation period (i.e., one day in this paper) is divided into many regulation movement periods. A regulation movement period is defined as the time spent between two neighbor zero-crossing points of frequency deviation. For evaluating the regulation performance for a regulation period (regulation movement) *j*, the following two values are applied.

Ratio of contributed regulating energy is:

13 $$\lambda _{{\mathrm{R}},j} = \frac{{E_{{\mathrm{R}},j}}}{{E_{{\mathrm{R - ideal}},j}}}.$$

Score of regulation correctness is defined as:

14 $$C_{{\mathrm{R}},j} = \left\{ \begin{array}{l}{\mathrm{1, }}\lambda _{{\mathrm{R}},j} > 0\\ {\mathrm{0, }}\lambda _{{\mathrm{R}},j} < 0\end{array} \right..$$

The regulation performance of the whole period can be evaluated by two indicators of effective regulation movement: average ratio of contributed regulating energy (*λ*_R-avg_) and the ratio of regulation correctness (*λ*_C_) as shown below.

15 $$\left\{ \begin{array}{l}\lambda _{{\mathrm{R - avg}}} = \frac{{\mathop {\sum}\limits_{j = 1}^{N_{\mathrm{R}}} {\lambda _{{\mathrm{R}},j}} }}{{N_{\mathrm{R}}}}\\ \lambda _C = \frac{{\mathop {\sum}\limits_{j = 1}^{N_{\mathrm{R}}} {C_{{\mathrm{R}},j}} }}{{N_{\mathrm{R}}}}\end{array} \right..$$

Here, *N*_R_ is the total amount of effective regulation movements of which the value of *E*_R-ideal_ of the movement exceeds 0.2 s (Supplementary Note 8 and Supplementary Fig. 20). More exactly, the payment scheme aims to focus on relatively large regulation movements by filtering the influence from small ones.

The final formula of contribution payment, *P*_contrib_., is

16 $$P_{{\mathrm{contrib}}{\mathrm{.}}} = \left( {0.8 \times \lambda _{{\mathrm{R - avg}}} + 0.2 \times \lambda _{\mathrm{C}}} \right) \times \frac{{P_{{\mathrm{m - rated}}}}}{{P_{{\mathrm{m - base}}}}}.$$

Here the component scalars are weighted as 80% and 20%, respectively. *P*_m-base_ is a base value for normalizing the payment, and here it is set to 42.19 MW for both HPPs to normalize the average payment of the unit in HPP 1 under Ep1 and S1 to 100.0 % (as in Table 1).

### Data availability

The data that support the findings of this study are available from the corresponding author upon reasonable request.

### Code availability

Relevant code may be rendered available from the corresponding author upon reasonable request.

## Electronic supplementary material

Supplementary Information
