# Supplementary material for: Burden on hydropower units for short-term balancing of renewable power systems
Source: Nat Commun. 2018 Jul 6;9:2633. doi: 10.1038/s41467-018-05060-4 (PMC6035178; doi:10.1038/s41467-018-05060-4)
Supplement: Supplementary file 1 — Supplementary Information [file 41467_2018_5060_MOESM1_ESM.pdf]

Burden on hydropower units for short-term balancing of renewable power systems

Supplementary Information

Supplementary Note 1

Kaplan turbines are installed throughout the world, and a feature of Kaplan turbines is the double-regulated mechanism: the runner blades (RBs) are automatically adjusted with the guide vane (GV) angle and head (on-cam relation) to achieve high efficiency over a wide operating range. In this paper, the off-cam operation refers to the case where the cam relation is violated, which causes extra efficiency loss. Kaplan turbines encounter serious wear, fatigue, and efficiency complications due to their flexible operation enabled by adjustable runner blades.

Supplementary Note 2

Nomenclature

Symbol	Unit	Description
a	[pu]	Runner blade angle
a_{tk}	[pu]	Runner blade angle at time step *tk*
b_{p}	[pu]	Governor droop
b_{p2}	[pu]	Governor droop of rest of units in the grid
b_{p3}	[pu]	Governor droop in Model 3
BL_{a}	[pu]	Runner backlash
BL_{gv}	[pu]	Guide vane backlash
C_R	[pu]	Score of regulation correctness
D	[pu]	Damping constant
D_{t}	[pu]	Turbine damping constant
E_{R}	[s]	Energy for regulation
$E_{\mathrm{R\text{-}ideal}}$	[s]	Ideal value of the energy for regulation
f	[pu]	Frequency or turbine rotational speed
f_{t}	[pu]	Frictional coefficient of tunnel
f_{p}	[pu]	Frictional coefficient of penstock
G	[pu]	Comprehensive gate opening
G_1	[pu]	Gain from frequency deviation to power deviation for the Kaplan unit
G_2	[pu]	Gain from frequency deviation to power deviation for the lumped hydropower plant
G_{F}	[pu]	Fitting function of the comprehensive gate opening
G_{g}	[pu]	Transfer function describing the grid
G_{P}	[pu]	Transfer function describing the head variation due to the discharge deviation in the penstock
G_{S}	[pu]	Transfer function describing the head variation due to the discharge deviation in the surge tank
G_{t}	[pu]	Transfer function describing the Francis turbine and waterway system
G_{PI}	[pu]	Gain from GVO deviation to frequency deviation for the PI controller
h	[pu]	Water head
h_0	[pu]	Initial water head
is	/	Indicator of sample number
K_1	[pu]	Scaling factor in Model 1
K_2	[pu]	Scaling factor of the lumped HPP in Model 2
K_3	[pu]	Scaling factor of the lumped HPP in Model 3
K_{d}	[s]	Governor parameter for the proportional term
K_{i}	[s^{-1}]	Governor parameter for the integral term
K_{p}	[pu]	Governor parameter for the derivative term
M	[s]	System inertia
M_{R}	[MW]	Regulation mileage
$M_{\mathrm{R\text{-}base}}$	[MW]	Base value of regulation mileage
N	/	Total amount of samples
N_{R}	/	Total amount of effective regulation movements of which the value of $E_{R\text{-}ideal}$ of the movement exceeds 0.2 s
p_{nl}	[pu]	Net load
p_{m}	[pu]	Active power
p_{m2}	[pu]	Active power of the lumped HPP
$p_{\mathrm{m,\ k}}$	[pu]	Active power at time step *k*
p_{m0}	[pu]	Initial active power
$P_{\mathrm{contrib.}}$	[pu]	Amount of contribution payment
P_{mile}	[pu]	Amount of mileage payment
P_{strength}	[pu]	Amount of strength payment
$P_{\mathrm{m\text{-}base}}$	[MW]	Base value of power for normalizing the contribution payment
$P_{\mathrm{m\text{-}rated}}$	[MW]	Rated power of generating unit
P_{step}	[MW]	Increase in output power caused by a frequency step change from 50 Hz to 49.9 Hz

q	[pu]	Discharge
q_0	[pu]	Initial discharge
s	[s^{-1}]	Complex variable in Laplace transform
S_R	[MW/Hz]	Regulation strength
S_{R1}, S_{R1-pu}	[pu]	Regulation strength of the Kaplan unit
S_{R2}, S_{R2-pu}	[pu]	Regulation strength of the lumped hydropower plant
S_{R-base}	[MW/Hz]	Base value of regulation strength
S_{RT}	[pu]	Regulation strength of all the units in the grid
t_p	[s]	Time constant in grid inverse model
t_k	/	Number of time step
T_{del-a}	[s]	Delay time in runner control
T_{del-gv}	[s]	Delay time in guide vane control
T_f	[s]	Period of frequency oscillation
T_r	[s]	Time constant of penstock elasticity
T_s	[s]	Time constant of surge
T_w	[s]	Water starting time constant
T_{wp}	[s]	Water starting time constant of penstock
T_{wt}	[s]	Water starting time constant of tunnel
T_y	[s]	Time constant of guide vane servo
T_{ya}	[s]	Time constant of runner servo
y	[pu]	Guide vane opening
$Y_{GV, dist}$	[pu]	Movement distance of guide vane
$Y_{RB, dist}$	[pu]	Movement distance of runner blade
α_p	[pu]	Elasticity coefficient of penstock
η	[pu]	Turbine efficiency
η_I	[pu]	Interpolation function of the turbine efficiency
η_{st}	[pu]	On-cam steady state efficiency
η_{Sj}	[pu]	Average value of the instantaneous efficiency during the operation period under a specific strategy (S_j)
λ_R	[pu]	Ratio of contributed regulating energy
λ_{R-avg}	[pu]	Average ratio of contributed regulating energy
λ_C	[pu]	Ratio of regulation correctness
$\Delta\eta$	[pu]	efficiency change
Δf	[pu]	Frequency deviation from set-point value
Δh	[pu]	Water head deviation from initial value
Δh_p	[pu]	Water head deviation from initial value due to hydraulic dynamics in penstock
Δh_s	[pu]	Water head deviation from initial value due to hydraulic dynamics in surge tank
$\Delta p_{m-ideal}$	[pu]	Ideal value of power deviation for the regulation
Δq	[pu]	Discharge deviation from initial value
Δt	[s]	Time step in simulation
Δy	[pu]	GVO deviation from set-point value

Acronyms

GV	Guide vane
GVO	Guide vane opening
HPP	Hydropower plant
PFC	Primary frequency control
PID	Proportional–integral–derivative
PI	Proportional–integral
PJM	PJM Interconnection Limited Liability Company
pu	Per unit
RB	Runner blade
RBA	Runner blade angle
SvK	Svenska Kraftnät (Swedish TSO)
TSO	Transmission system operator
VRE	Variable renewable energy

The symbols and acronyms in this supplementary are only introduced here in the Nomenclature.

Supplementary Note 3

Much research has been conducted on modelling and dynamic processes of hydro turbines[1-6], HPPs[7-12], and pumped storage plants[13,14], and many meaningful achievements in control of HPP have been introduced[15]. These include detailed models of turbine and waterway systems with a focus on Francis turbines. Various Kaplan turbine control models have been proposed[16,17], and their transients and interactions with power system have been investigated[18-21]. However, hydraulic dynamics that affect the net head across the turbine are normally simplified, *e.g.* surge tank modelling is ignored. Hence in this study, we build a comprehensive HPP model with a Kaplan turbine by applying MATLAB/Simulink that includes a waterway system.

The overall structure of the model is shown in Supplementary Figure 1. All the variables in this paper are introduced in Nomenclature in Supplementary Note 2. The open loop "hydropower plant" model with the red dashed outline is Model 1, for simulating the efficiency (η), power output (p_{m}), GVO and RBA. The closed loop model for the Nordic power system with the blue dashed outline is Model 2, for simulating the frequency quality. Model 1 and Model 2 correspond to the two models in Figure 2. The governor system and the turbine are modelled in detail, considering the water head variation due to the hydraulic dynamics in waterway system. These components of the system are introduced in the following content. The per-unit (pu) system is adopted for describing all the models. The scaling factors, K_1 and K_2, are introduced in Supplementary Note 6.

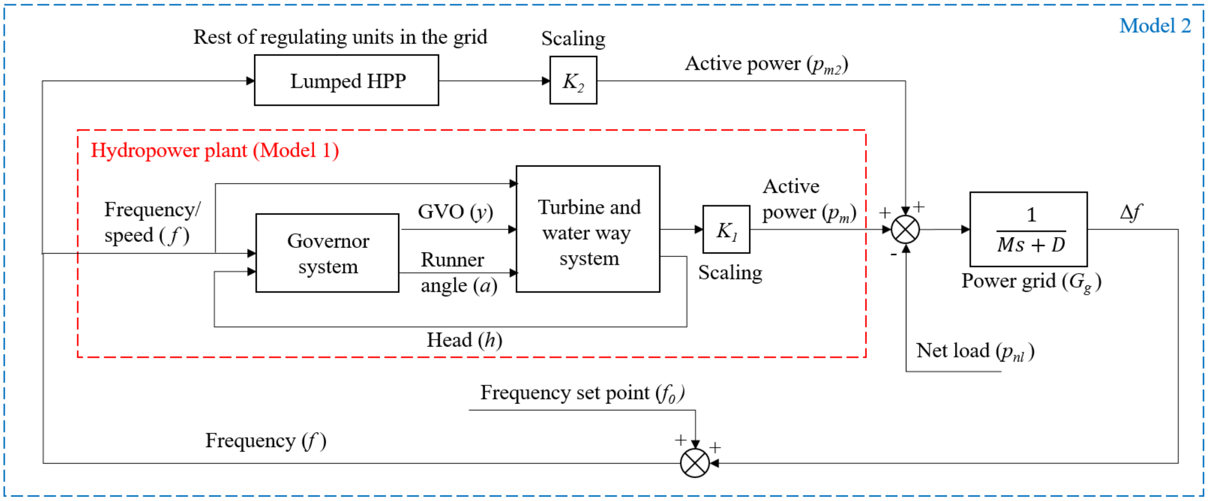

Supplementary Figure 1. Overall model structure of a hydropower system with a Kaplan turbine in the Nordic power system. Some detailed set points and feedback signals are omitted here but included in the more detailed block scheme shown in the following content.

Kaplan turbine and waterway system

The model of Kaplan turbine and waterway system is shown in Supplementary Figure 2.

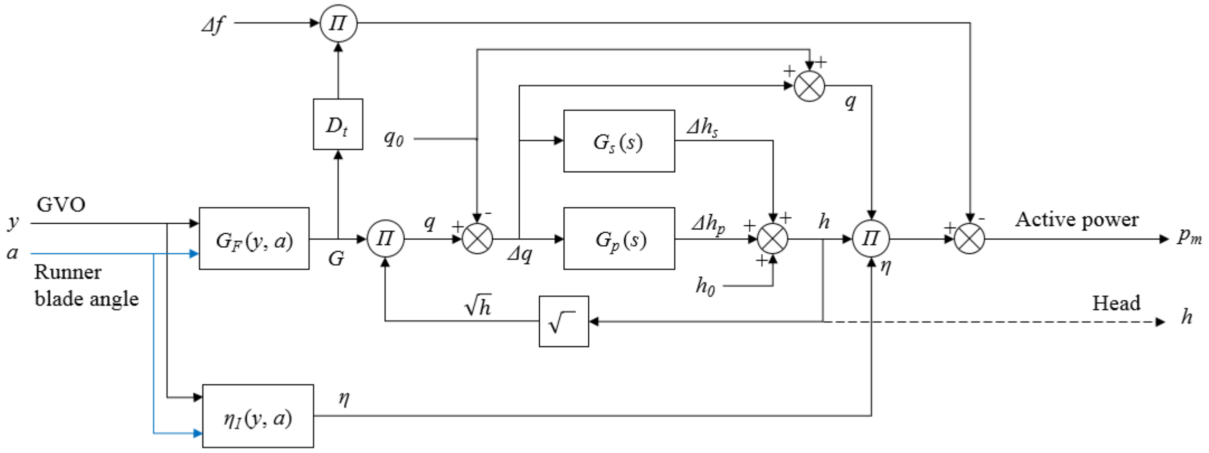

Supplementary Figure 2. Block diagram of a model of Kaplan turbine and waterway system (in Model 1). The signal of runner blade angle is presented in blue, for distinguishing from the GVO signal

The active power from the turbine is described by the classical simplified non-linear model[1,22].

$$q = q_0 + \Delta q = G\sqrt{h} = G\sqrt{h_0 + \Delta h}\ , \tag{1}$$

$$p_m = \eta q h = \eta G h^{3/2}\,. \tag{2}$$

Supplementary Equations (1) and (2) are for single-regulated turbines, *e.g.* Francis turbines. While for the double-regulated turbine in this study, G is introduced as a comprehensive gate opening that is identified from the values of GVO (y) and runner blade angle (a), as shown in a fitting function

$$G = G_F\left(y, a\right). \tag{3}$$

The efficiency value is obtained from an interpolation function

$$\eta = \eta_I\left(y, a\right). \tag{4}$$

These two functions need to be achieved from measurement data that is presented in Supplementary Note 4.

The head is affected by hydraulic dynamics from the elastic penstock, draw water tunnel and surge tank [1,23], as described in

$$\begin{cases} h = h_0 + \Delta h \\ \Delta h = \Delta h_{\mathrm{p}} + \Delta h_{\mathrm{s}} \end{cases}. \tag{5}$$

In frequency domain, the transfer function describing the head variations due to discharge deviations in the penstock is

$$\frac{\Delta h_{\mathrm{p}}}{\Delta q} = G_p\left(s\right) = -\frac{T_{\mathrm{wp}}}{T_{\mathrm{r}}}\cdot\frac{\alpha_{\mathrm{p}} T_{\mathrm{r}} f_{\mathrm{p}} s^2 + T_{\mathrm{r}} s + f_{\mathrm{p}}}{1 + \alpha_{\mathrm{p}} T_{\mathrm{r}}^2 s^2}\,. \tag{6}$$

The transfer function describing the head variations due to discharge deviations in the surge tank is

$$\frac{\Delta h_{\mathrm{s}}}{\Delta q} = G_{\mathrm{s}}\left(s\right) = -\frac{T_{\mathrm{wt}} s + f_{\mathrm{t}}}{T_{\mathrm{wt}} T_{\mathrm{s}} s^2 + T_{\mathrm{s}} f_{\mathrm{t}} s + 1}\,. \tag{7}$$

When turbine damping (D_{t})[1] is considered, the equation of the active power becomes

$$p_m = \eta G h^{3/2} - D_t G \Delta f\ . \tag{8}$$

Governor system with filters

The model of a governor system for a double-regulated Kaplan turbine is shown in Supplementary Figure 3. Except for a standard PID (proportional–integral–derivative) controller with droop, common mechanical components and a 2-D (two-dimensional) lookup table, we also include the artificial filter for the signal of RBA. The filter for runner blade angle is a floating dead zone (or floating dead band)[24,25], which is equivalent to the backlash[26] in the Simulink model. The implementations of runner relief strategy S2 and S3 are based on the setting of the filter.

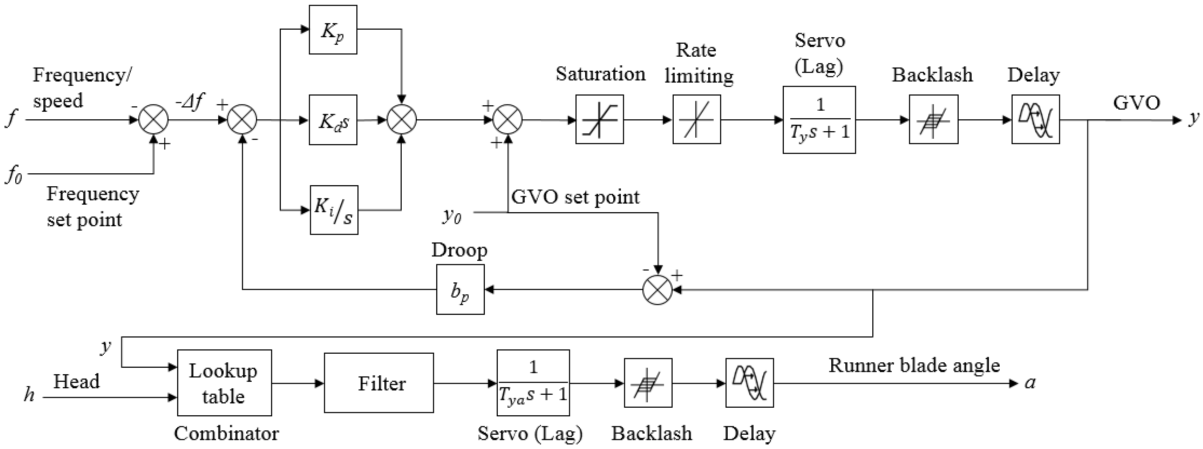

Supplementary Figure 3. Block diagram of governor system of the double-regulated Kaplan turbine

Simplified power grid

In order to investigate the frequency quality under different operation strategies, a simplified single-area model[27,28] representing the Nordic power grid is included in Supplementary Figure 1 and described in Supplementary Equation (9). The identified parameters[27,28] of the Nordic power grid are presented in Supplementary Table 1. The base power of the system is 37650 MW[28].

$$\text{Grid: } G_g(s) = \frac{1}{Ms + D} \tag{9}$$

Supplementary Table 1. Parameters for the Nordic power grid model

Symbol	Parameter	Value
M	System inertia	14.6 [s]
D	Damping constant	0.66 [pu]

Lumped HPP

The lumped HPP represents the rest of regulating units in the power grid, by assuming that all the regulation in the grid is provided by hydropower. As shown in **Supplementary Figure 4**, the model contains a governor and a simplified representation of a Francis turbine and waterway system that is described in

$$G_{\mathrm{t}}(s) = \frac{-T_{\mathrm{w}}s + 1}{0.5T_{\mathrm{w}}s + 1}. \tag{10}$$

Here the value of T_{w} is set to 1.5 s. The values of mechanical components in the governor adopt the values

in HPP 1 (Supplementary Table 4).

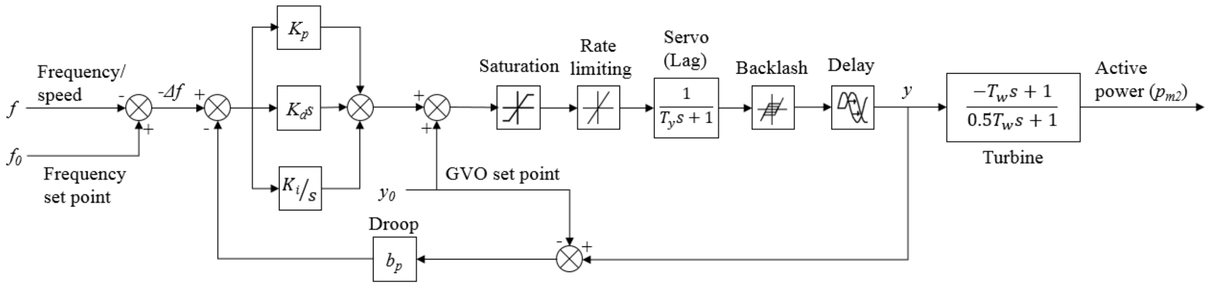

Supplementary Figure 4. Block diagram of a model of lumped HPP. The model contains a governor and a simplified representation of Francis turbine.

Net load

Net load can be concisely interpolated as the difference between gross load and VRE generation[29], and its variability combines variability in power consumption and variable power generation that needs to be balanced by regulating power in power systems[30]. It is a key for studies on feasibility of renewable energy systems.

For the Nordic power system, an explanatory equation for the net load [30] is written as

$$\text{Net load} = \text{Load} - \text{VRE} - \text{thermal} - \text{nuclear} = \text{Hydro} + \text{import} - \text{export} \tag{11}$$

In this paper, we investigate the deviation value of the net load and the grid frequency, and we ignore the power import/export and variations from thermal power and nuclear power. Hence the net load variation directly corresponds to the regulating power variation from hydropower units, as shown in

$$\Delta\,\text{Net load} = \Delta\,\text{Load} - \Delta\,\text{VRE} = \Delta\,\text{Hydro} \tag{12}$$

As shown in Supplementary Figure 2, Supplementary Figure 14 and Figure 2 in the main body of this paper, the net load is the input of Model 2 and the output of Model 3. The detailed method of computing the net load is presented in Supplementary Note 6, and a demonstration is shown in Supplementary Figure 15.

Supplementary Note 4

- Comprehensive gate opening and efficiency from measurements

A common approach for building numerical models of hydro turbines is by applying the turbine characteristics curves. A limitation of that method is that the characteristics curves, when obtained from physical turbine model tests, might lead to deviations from the actual performance in real HPPs. Thus in this study, we develop the turbine characteristics regarding the comprehensive gate opening and efficiency of both HPPs from on-site measurement data.

In addition to the standard model acceptance tests[31] for evaluating the turbine characteristic, in HPP 1 and HPP 2, specific on-site index tests[32,33] were conducted for a better combination between GVO and RBA to achieve higher efficiency. The measured scatter data of comprehensive gate opening and efficiency can be calculated for a limited operating region and applied for the fitting or the interpolation. Through the surface fittings, the characteristics of the turbine for a larger operation range can be obtained. In order to achieve a relatively higher efficiency accuracy demanded by this study, the interpolation of the efficiency data is adopted. However the measurement data are only available in a certain range, hence extra data for larger operating points are added from the fitting data for the extrapolation.

Fittings

The comprehensive gate opening and efficiency for each HPP are fit to a quadratic polynomial surface using

$$G = G_F\left(y,a\right) = p_{\mathrm{G00}} + p_{\mathrm{G10}}a + p_{\mathrm{G01}}y + p_{\mathrm{G20}}a^2 + p_{\mathrm{G11}}ay + p_{\mathrm{G02}}y^2; \tag{13}$$

$$\eta = \eta_{\mathrm{F}}\left(y,a\right) = p_{\eta 00} + p_{\eta 10}a + p_{\eta 01}y + p_{\eta 20}a^2 + p_{\eta 11}ay + p_{\eta 02}y^2. \tag{14}$$

where, p_{Gij} and $p_{\eta ij}$ are the coefficients of the fitting. The fittings are shown in Supplementary Figure 5 through Supplementary Figure 7. The fitting accuracy is of great importance, especially for the efficiency because it directly affects the results of efficiency losses. The reason for choosing the quadratic polynomial surface can be interpreted as following: (1) The overall distribution law of the efficiency is close to the realistic characteristic that is normally demonstrated as the hill chart. This is mainly important for extrapolation because other higher order fitting methods only can achieve slightly better interpolation but terrible extrapolation; (2) A relatively accurate approximation can be achieved. The residual range for HPP 1 is within ±0.6 % and mostly within ±1.0 % for HPP 2, as shown in Supplementary Figure 6 (b) and Supplementary Figure 7 (b); (3) This fitting type is simple and concise. It is worth stating that the fitting of an operating point far from the on-cam range might not be accurate, however, this will not affect the results of this study because the fitting is only applied for small disturbance simulation in this study.

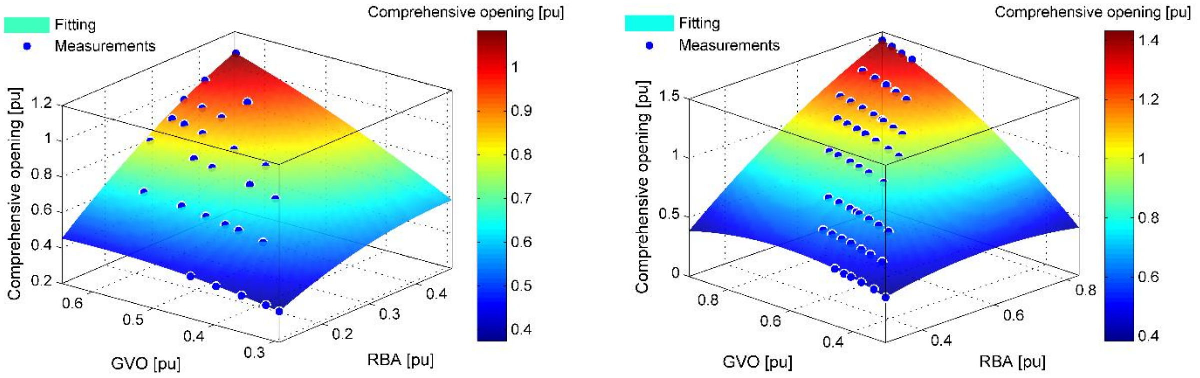

(a) **(b)**

Supplementary Figure 5. Fitting of data of comprehensive gate opening G_F for (a) HPP 1 and (b) HPP 2

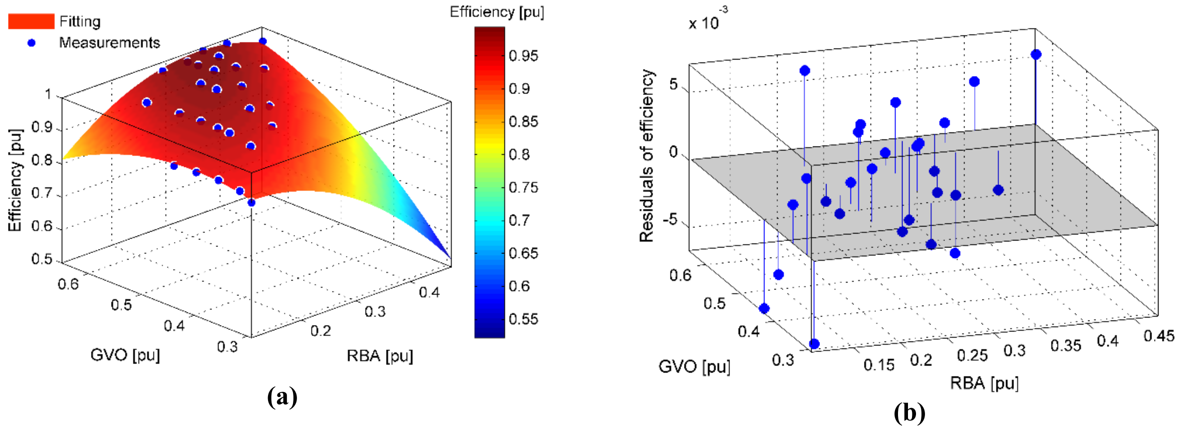

(a) **(b)**

Supplementary Figure 6. (a) Fitting and (b) residuals of turbine efficiency data of HPP 1. The efficiency value is normalized with respect to the maximum efficiency value.

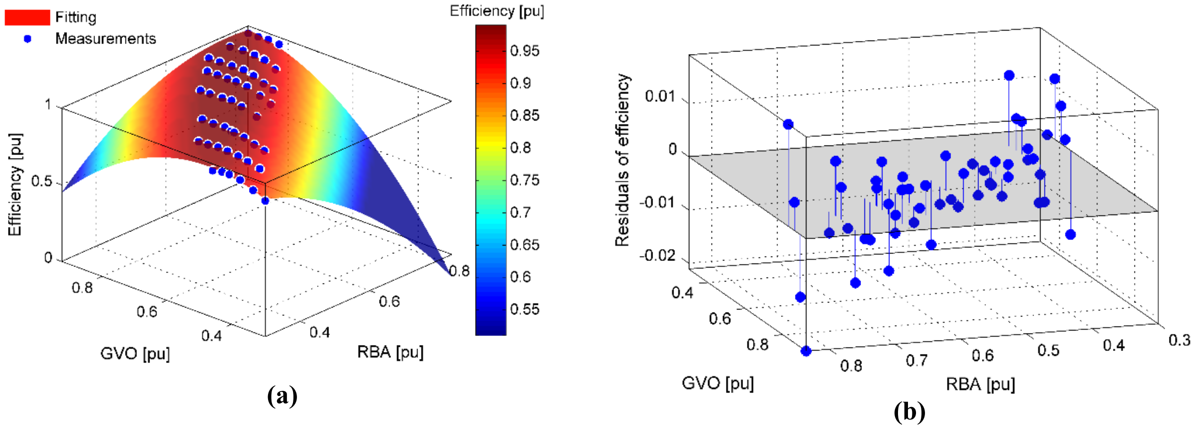

(a) **(b)**

Supplementary Figure 7. (a) Fitting and (b) residuals of turbine efficiency data of HPP 2. The efficiency value is normalized with respect to the maximum efficiency value.

Interpolations and extrapolation of efficiency

We apply piecewise cubic interpolation to obtain the final efficiency data (Supplementary Figure 8). By adopting the added points (in red) from the fitting data, the small operation range covered by the index tests is extended, then the efficiency data can support all the small disturbance simulations in this paper.

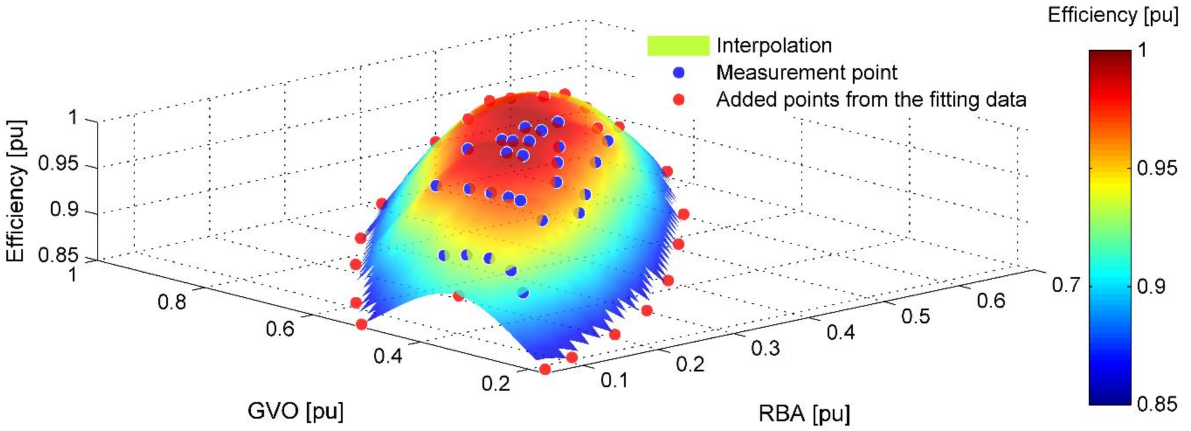

(a)

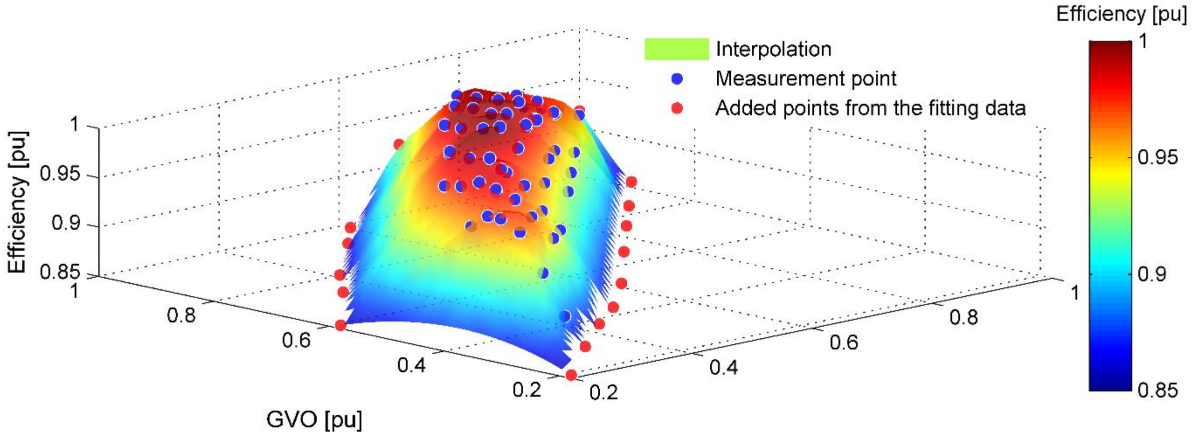

(b)

Supplementary Figure 8. Interpolation of turbine efficiency data of HPP 1 (a) and HPP 2 (b). The blue scatters are from the measurements in the index test, corresponding to the dots in Supplementary Figure 5 through Supplementary Figure 7; the red scatters are extracted from the fitting for extrapolation.

Supplementary Note 5

- Model validation: comparison of measurements and simulations

We compare the simulations with the on-site measurements of HPP 1 for identifying the mechanical characteristics (servo, backlash, and delay) and for verifying the model. Part of the measurement devices are demonstrated in Supplementary Figure 9.

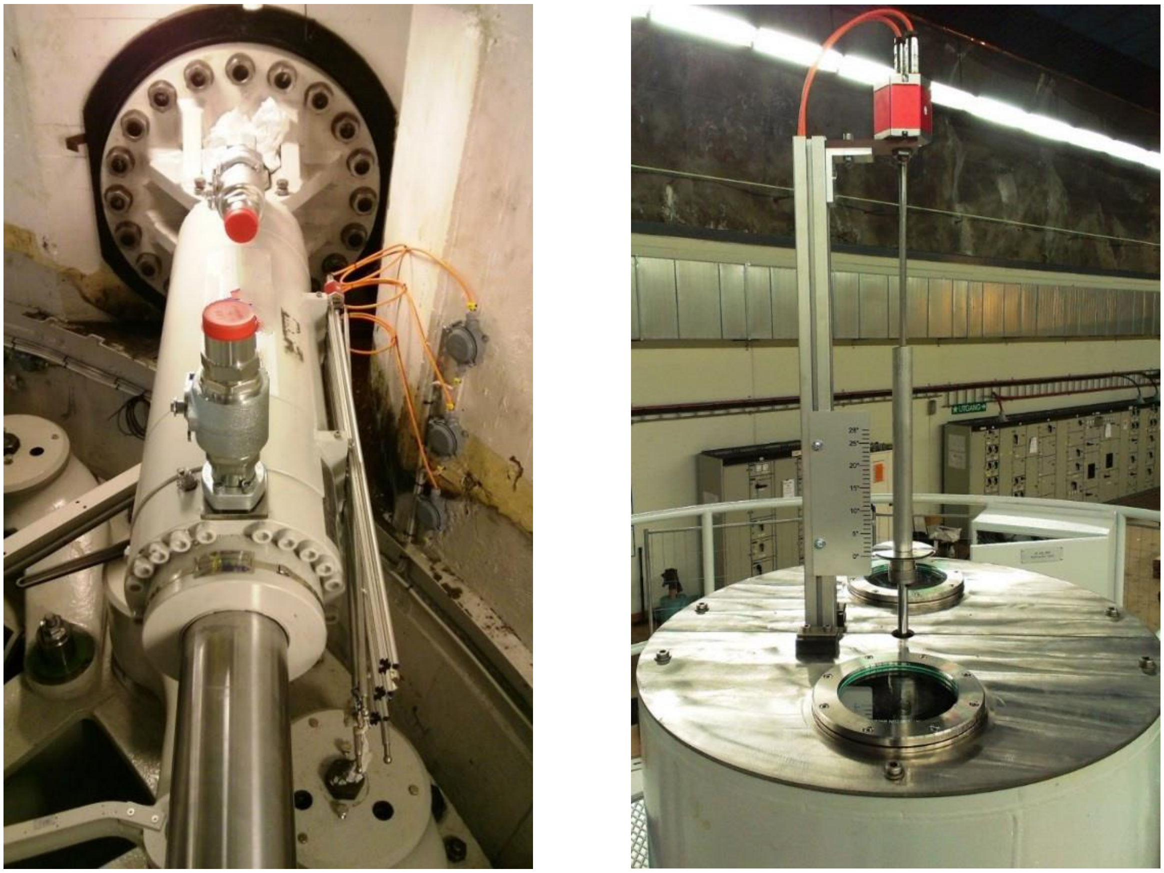

(a) **(b)**

Supplementary Figure 9. Part of measurement devices in HPP 1. (a) Guide vane servo with feedback transducer, connected with orange cables, on its right. (b) Feedback device for runner blade angle. The red device on top is the transducer. The rod is close to 14 m long and ends in the runner hub. The visual scale is in degrees.

Identification of the mechanical characteristics

The representative values of the mechanical characteristics (*i.e.* servo, backlash and delay in Supplementary Table 4) in the governor system are unknown at first, and they are important for the simulation of the wear indicators and the efficiency loss. Therefore, a specific identification of the values is conducted based on various sinusoidal input fed into the governor system from a signal generator, and the output values (*e.g.* GVO, RBA) are measured and compared with simulations. The system during the tests is illustrated in Supplementary Figure 10. Then the frequency-domain response of the governor system is achieved (Supplementary Figure 11 and Supplementary Figure 12). Under the proper setting of the values of the mechanical characteristics (Supplementary Table 4), the simulation matches the measurement well.

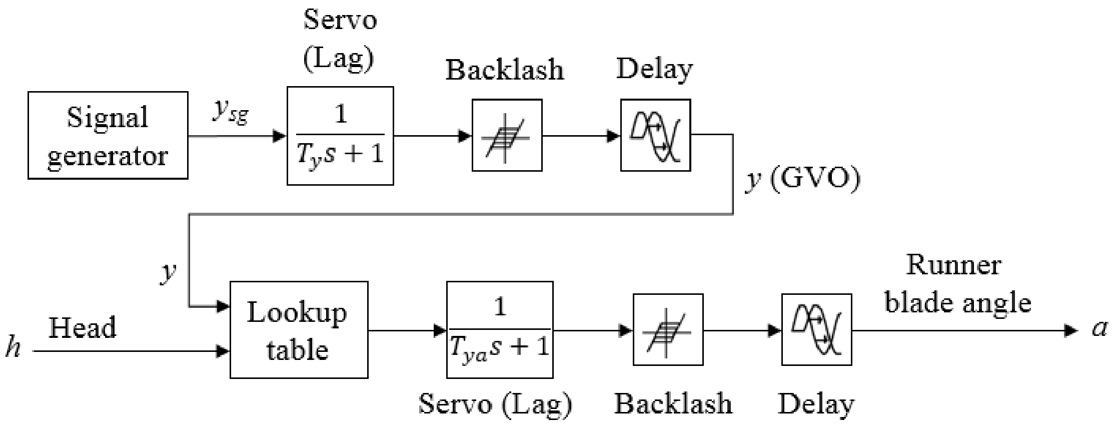

Supplementary Figure 10. Block diagram of the mechanical system in the governor during the tests for identification of the mechanical characteristics. Different sinusoidal input (y_{sg}) is fed into the system from a signal generator, and the response of the GVO and the RBA are measured.

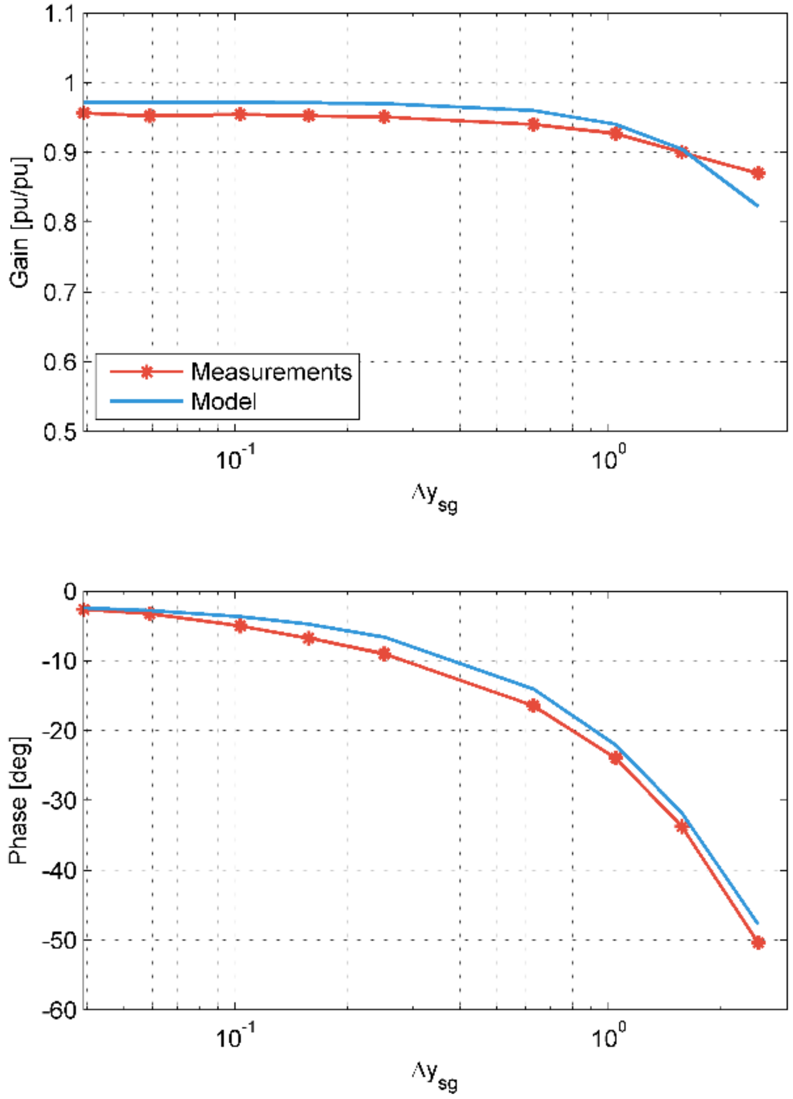

Supplementary Figure 11. Bode-like plot for $\Delta y_{sg} \rightarrow \Delta y$, HPP 1. The amplitude in y_{sg} is 0.01 pu. In the simulation model, $T_y = 0.25$ s, $T_{del\text{-}gv} = 0.097$ s, $BL_{gv} = 0.00029$ pu.

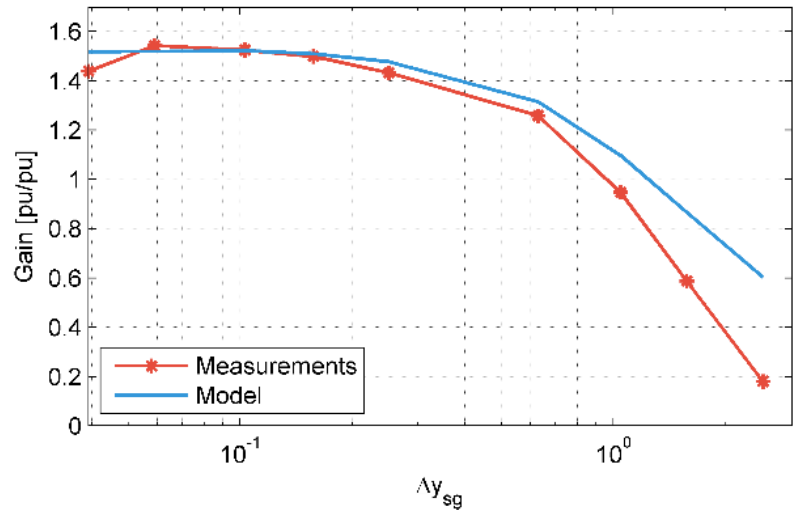

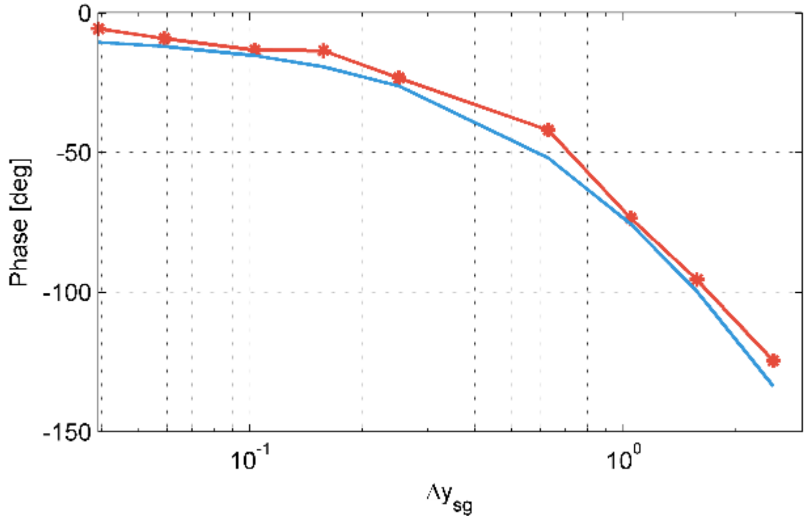

Supplementary Figure 12. Bode-like plot for $\Delta y \rightarrow \Delta \alpha$, HPP 1, with $T_{y\alpha} = 0.9$ s, $T_{\text{del-}\alpha} = 0.41$ s, and $BL_{\alpha} = 0.0013$ pu. The amplitude in y_{sg} is 0.01 pu, so the amplitude in y is close to 0.0095 pu, deduced from the gain in Supplementary Figure 11.

Comparison under normal PFC

For HPP 1, the simulation of the dynamic processes of the hydropower system under normal PFC is compared to the measurements (Supplementary Figure 13), under the governor parameters Ep1 (Supplementary Table 2). The simulation of the GVO, the RBA and the power output has a good agreement with the measurements, showing that the model can yield trustworthy simulation results.

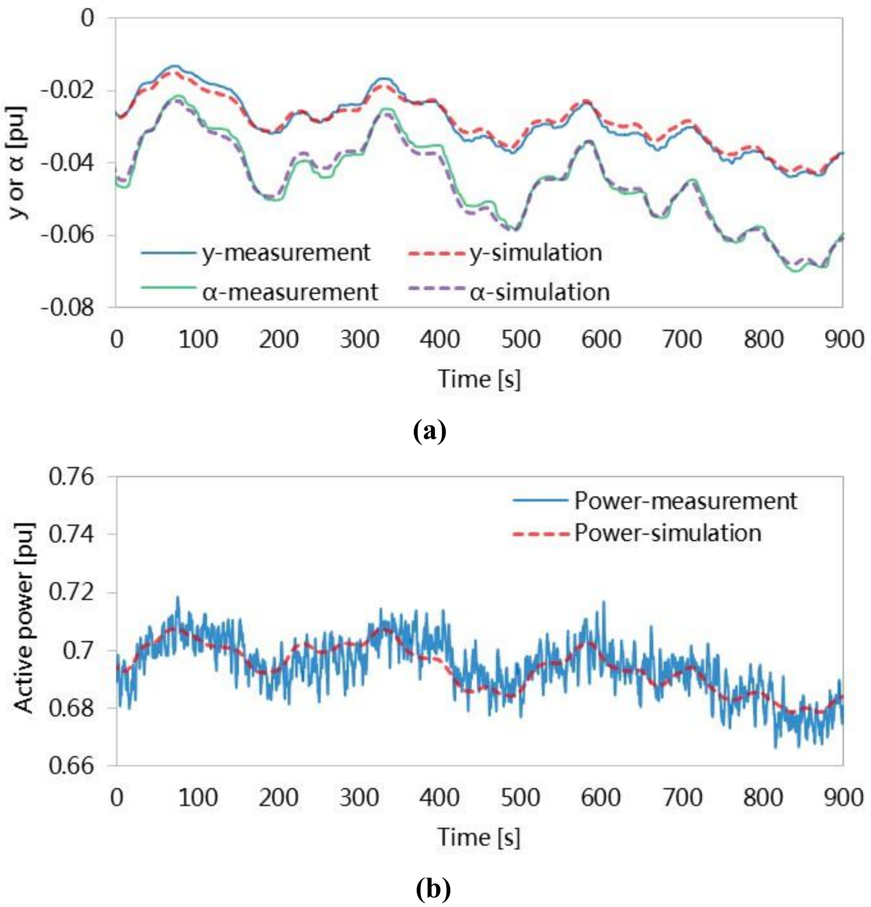

Supplementary Figure 13. Comparison of measurement and simulation during a period of normal PFC. (a) Guide vane opening, y, and runner blade angle, α, the deviation value is shown. (b) Active power.

Supplementary Note 6

Overall setting of simulations

The time-step of all the simulations is 0.02 s. The input signal of Model 1 and Model 3 is a sequence of measured one-day (24-hour) Nordic grid frequency, and its sampling time is 1.0 s. The implementation of the strategy S2 and S3 in the simulations is by setting the value of the filter that is the floating dead zone for RBA. For the S2 and S3, the value of the floating dead zone is set to 0.03 pu and 1.0 pu respectively; while for the S1, this filter value is set to 0. For the ideal case S0, all the mechanical components in runner blade part (after the combinator in Supplementary Figure 3) are removed to achieve a purely on-cam operation. The initial steady-state value of water head is set to the rated value (1.0 pu).

Settings for simulating the GVO, RBA and the efficiency loss

The adopted model is the open loop HPP model (Model 1) in Supplementary Figure 1, without the engagement of the power grid components. Hence the scaling factor (K) is set to 1.0. As presented in Figure 2, there are 168 (3×4×2×7) simulation cases conducted. However, fewer simulation cases are needed for analysis of some indicators. For example, only three simulation cases for GVO are actually needed (for the parameter set Ep1~Ep3), because the GVO is not influenced by other settings (strategy, HPP and operating point) in our simulation.

Simulation of the net load and the frequency sequence

In a previous work[24], a method of simulating and evaluating the frequency quality for units with Francis turbine is introduced. Here, the method is improved and extended for double-regulated Kaplan turbines.

(1) Simulation of the net load
As shown in Figure 2, new frequency sequences are simulated by Model 2 (Supplementary Figure 1) based on a certain net load. This unknown sequence of one-day net load is calculated from the original measured frequency, by a "grid inverse" model[27] shown in Supplementary Equation (15) and Supplementary Figure 14. A pole with time constant t_p is applied for avoiding high amplification of high frequency noise in the grid frequency signal, based on the theory of internal model control[34]. Its value is set to 0.1 in this study. The values of M and D are found in Supplementary Table 1.

$$\text{Grid inverse: } G_{gi}(s) = \frac{Ms + D}{t_p s + 1} \tag{15}$$

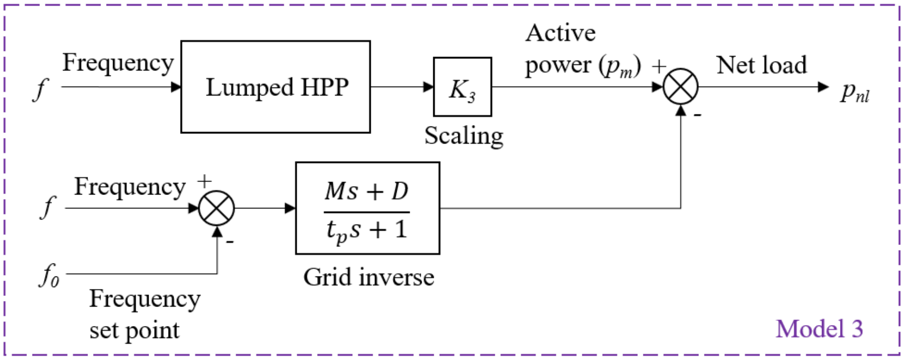

Supplementary Figure 14. Block diagram of Model 3 with the "grid inverse" model for computing the net load; the lumped hydropower plant block is described in the part with dashed outline in Supplementary Figure 1.

In Model 3, all the regulating HPPs in the Nordic power grid are lumped into one scaled HPP with the scaling factor (K_3). The model of the lumped HPP is the same in **Supplementary Figure 4**. Currently, the Nordic TSOs require a fixed amount of regulating power of PFC in the whole grid: 753MW for 0.1 Hz frequency deviation, hence the total regulation strength is 7530 MW/Hz, and it is normalized as S_{RT} that is 10.0 pu[27]. Consequently, in Model 3, the product of the governor static gain $1/b_p$ (in Methods) and K_3 is 10.0 pu[27]. Here, the governor parameter set is selected as Ep1 for computing the net load, and the droop in Model 3 is noted as b_{p3} (b_{p0} = 0.04). Therefore, the value of K_3 is set to 0.4 pu (10×b_{p3} pu).

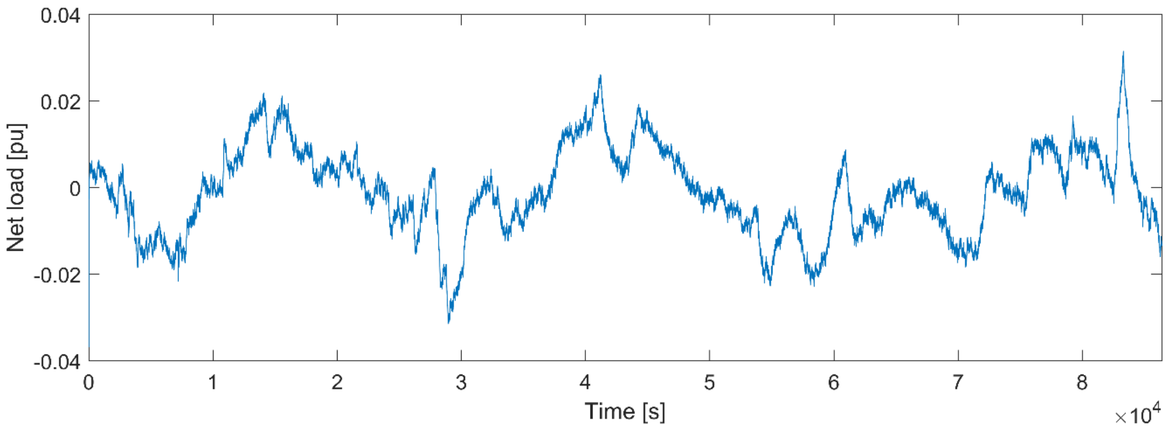

Supplementary Figure 15. Demonstration of the one-day sequence of net load (deviation value in per unit)

(2) Simulation of the frequency sequence

With the simulated net load (Supplementary Figure 15) as input, Model 2 is applied to simulate new frequency sequences. In Model 2, the regulation power in the grid is provided by the Kaplan unit (Model 1) that is the examining object as well as the rest of units (Francis unit in the lumped HPP). The influence of the regulation from the Kaplan unit on the whole grid is investigated. In Model 2, the values of two scaling factors (K_1 and K_2) are described in

$$\begin{cases} S_{\mathrm{R1}} + S_{\mathrm{R2}} = S_{\mathrm{RT}} \\ S_{\mathrm{R1\text{-}pu}} + S_{\mathrm{R2\text{-}pu}} = G_1 K_1 + G_2 K_2 = S_{\mathrm{RT\text{-}pu}} \end{cases} . \tag{16}$$

Here the S_{R1} and S_{R2} are the regulation strength of the Kaplan unit and the lumped HPP respectively; their corresponding values in per unit are represented as $S_{\mathrm{R1\text{-}pu}}$ and $S_{\mathrm{R2\text{-}pu}}$. The simulated regulation strength of the Kaplan turbine under different conditions are shown in Supplementary Table 6. G_1 and G_2 are the gains from frequency deviation to power deviation for the Kaplan unit and the lumped HPP, as presented in

$$\begin{cases} G_1 = \dfrac{S_{\mathrm{R1}}}{10\Delta f_{\mathrm{step}} \cdot P_{\mathrm{m\text{-}rated}}} \\ G_2 = \dfrac{1}{b_{\mathrm{p2}}} \end{cases}, \text{[pu]}. \tag{17}$$

Here, Δf_{step} (0.1 Hz) is the step change value of frequency that corresponds to 0.002 pu; $P_{\mathrm{m\text{-}rated}}$ is the rated power of the Kaplan unit, b_{p2} means the droop in the lumped HPP. For example, for HPP 1 under the Ep1 setting, the values of K_1 and K_2 are 0.00138 and 0.3977; for HPP 2 under the Ep1 setting, the values of K_1 and K_2 are 0.00112 and 0.3980.

In this paper, we keep the regulation strength S_{R2} as constant. The S_{R1} of the Kaplan turbine changes with various conditions and influences the frequency quality. For each HPP, the new frequency sequences are compared to the frequency sequence from Ep1 and S1 (Supplementary Table 5). The operating point has little influence on the frequency quality, hence the operating point stays the same (selected as point 5) in all simulations, as shown in Figure 2.

Supplementary Table 2. Standard controller parameters in Vattenfall HPPs

Parameter	Ep0	Ep1	Ep2	Ep3
b_p (or E_p)	0.1	0.04	0.02	0.01
K_p	1	1	1	2
K_i	1/6	5/12	5/6	5/3

Supplementary Table 3. Basic information of a generating unit of HPP 1 and HPP 2

Parameter	HPP 1	HPP 2
Rated power (p_{m0}) [MW]	52.0	42.3
Rated water head (h_0) [m]	30	24
Rated discharge (q_0) [m^3/s]	170	180
Rated rotation speed [r/min]	150	125
Number of runner blades	5	5

Supplementary Table 4. Parameter values of the HPP 1 and HPP 2 for simulation settings. There is no surge tank in HPP 2, hence the values of T_{wt}, T_s and f_t are shown as N/A.

HPP 1				HPP 2			
Parameter	Value	Parameter	Value	Parameter	Value	Parameter	Value
T_y	0.25 s	T_{wt}	13.2 s	T_y	0.25 s	T_{wt}	N/A
T_{ya}	0.90 s	T_r	0.1 s	T_{ya}	0.90 s	T_r	0.07 s
$T_{del\text{-}gv}$	0.097 s	T_s	350.0 s	$T_{del\text{-}gv}$	0.097 s	T_s	N/A
$T_{del\text{-}a}$	0.410 s	α	0.33	$T_{del\text{-}a}$	0.410 s	α	0.33
BL_{gv}	0.00029 pu	f_t	$0.0065\times q_0$	BL_{gv}	0.00029 pu	f_t	N/A
BL_a	0.00132 pu	f_p	$0.0120\times q_0$	BL_a	0.00132 pu	f_p	$0.010\times q_0$
T_{wp}	1.7 s	D_t	0	T_{wp}	1.01 s	D_t	0

Supplementary Table 5. Detailed simulation results of frequency quality

Para.	Stragegy	HPP 1				HPP 2			
		Mean [Hz]	Std [Hz]	RMSE [Hz]	ΔRMSE	Mean [Hz]	Std [Hz]	RMSE [Hz]	ΔRMSE
Ep1	S1	0.00886	0.04910	0.04989	0	0.00935	0.04912	0.05000	0
	S2	0.00887	0.04914	0.04993	0.07%	0.00935	0.04914	0.05002	0.04%
	S3	0.00888	0.04924	0.05004	0.29%	0.00936	0.04923	0.05012	0.23%
Ep2	S1	0.00883	0.04884	0.04963	-0.53%	0.00931	0.04890	0.04978	-0.44%
	S2	0.00883	0.04887	0.04966	-0.48%	0.00931	0.04892	0.04980	-0.41%
	S3	0.00883	0.04913	0.04992	0.05%	0.00931	0.04913	0.05001	0.02%
Ep3	S1	0.00876	0.04834	0.04913	-1.54%	0.00924	0.04847	0.04934	-1.31%
	S2	0.00876	0.04836	0.04915	-1.49%	0.00924	0.04849	0.04936	-1.27%
	S3	0.00870	0.04891	0.04968	-0.43%	0.00915	0.04892	0.04977	-0.45%

In the column "Mean", the deviations of the frequency mean values from the rated value (50 Hz) are shown. "Std" stands for the standard deviation. "RMSE" is the root mean square error with respect to the rated frequency. The values in the column "ΔRMSE" are the relative changes of the RMSE compared to the value under Ep1 and S1 for each HPP. A negative value of "ΔRMSE" in blue means the decrease of the RMSE, indicating a better frequency quality. While in Table 1, the signs of the values are changed, making a negative value to represent a decrease of frequency quality. The difference of the values under various conditions shows the influence of the single Kaplan unit on the frequency of the whole grid.

Supplementary Table 6. Simulated regulation strength (in MW/Hz) of the Kaplan turbine under different conditions.

Para.	Operation point	HPP 1			HPP 2		
		S1	S2	S3	S1	S2	S3
Ep1	1	41.08	31.59	13.29	34.41	28.18	9.27
	2	42.77	33.35	14.15	37.84	31.34	11.62
	3	44.38	35.43	15.36	37.05	30.52	10.28
	4	44.35	35.56	14.91	36.30	28.50	11.58
	5	42.50	33.94	15.02	37.51	31.56	13.44
	6	43.97	35.51	14.96	37.85	31.83	12.71
	7	46.60	38.22	14.74	38.80	33.67	12.96
	Average	43.66	34.80	14.63	37.11	30.80	11.69
Ep2	1	85.79	78.25	21.42	74.17	67.50	11.13
	2	84.74	77.73	23.79	72.82	66.49	10.82
	3	85.65	78.65	25.09	73.49	65.20	15.93
	4	87.39	80.98	24.77	73.66	67.57	15.65
	5	89.81	84.10	24.74	75.93	70.54	15.93
	6	94.55	90.21	23.89	77.07	72.27	17.33
	7	97.53	94.24	23.12	73.06	70.07	15.85
	Average	89.35	83.45	23.83	74.31	68.52	14.66
Ep3	1	178.41	178.77	23.86	147.80	139.73	-4.48
	2	181.89	182.75	21.86	145.90	139.70	-2.97
	3	184.76	185.16	19.46	149.45	143.44	-5.21
	4	186.19	186.15	17.77	143.64	140.83	-7.39
	5	185.30	185.06	17.60	140.90	139.10	-7.09
	6	186.41	186.21	16.64	141.42	139.76	-4.32
	7	186.64	186.71	16.12	144.22	142.29	-0.10
	Average	184.23	184.40	19.04	144.76	140.69	-4.51

The frequency step change is from 50 Hz to 49.9 Hz. The average values of seven operating points are shown in the row "Average", and they correspond to the values in Table 1. Positive and negative values are shown with green and red bars respectively.

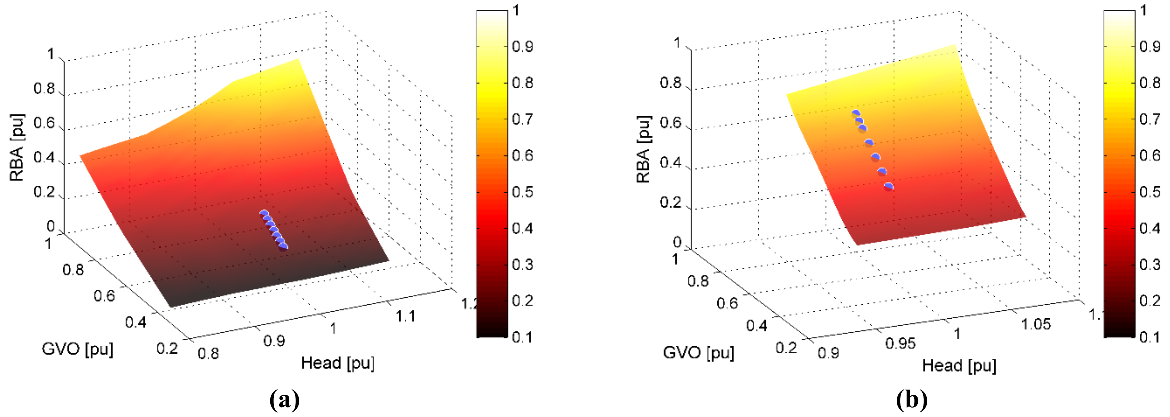

Supplementary Figure 16. Illustration of the combinator table for the turbine in the HPP 1 (a) and HPP 2 (b). In each figure, seven on-cam operating points are highlighted by blue scatters, they are within the maximum efficiency range.

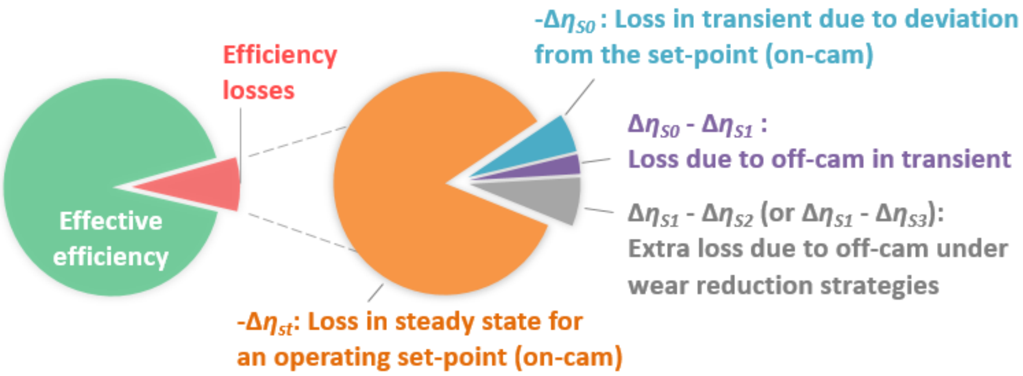

Supplementary Figure 17. Compositions of efficiency losses analyzed in this study for Kaplan turbines

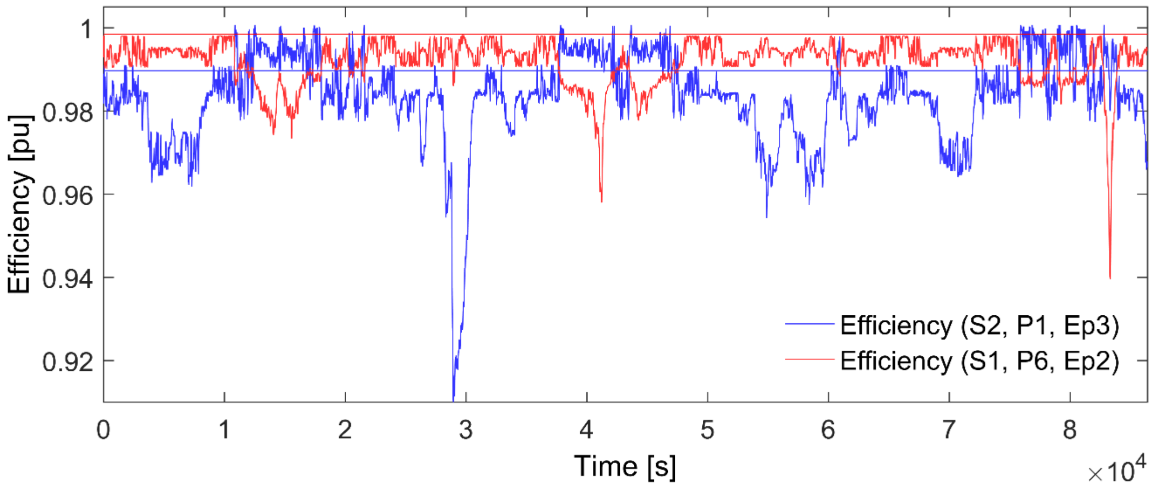

Supplementary Figure 18. Instantaneous efficiency during the one-day (86400 s) operation under different settings. The blue straight line and the red straight line show the on-cam steady state efficiency of point 1 and point 6 respectively.

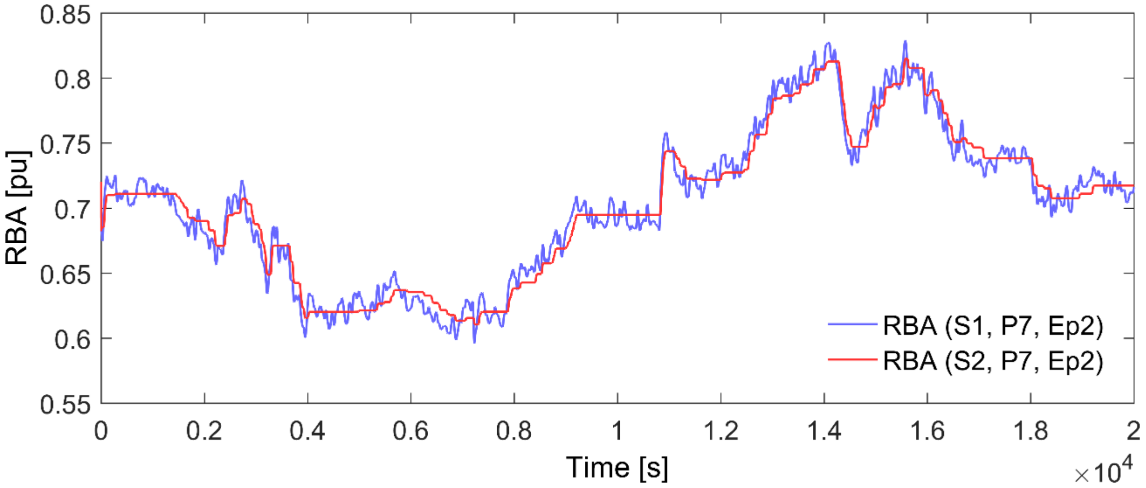

Supplementary Figure 19. Demonstration of the reduction of RB movements under operation strategy S2, comparing to the normal frequency control case (S1)

Supplementary Note 7

- Time of commitment and determination of payments

The time of commitment and determination of payments for the cases in different countries are introduced briefly here.

(1) Commitment of payments
For the TSO SvK in Sweden, bids are placed one and two days ahead for each hour of a 24-hour period[35].

For the PJM Regulation Market in the USA: cost-and price-based regulation offers (*i.e.*, commitments) and any applicable cost information must be supplied by 14:15 the day prior to operation, and are applicable for the entire 24-hour period for which they are submitted[36]. PJM settles ancillary service transactions in its day-ahead markets on an hourly basis.

In the case in China (the case in the revision B above in green), the compensation is an award of regulation contribution and is determined solely by the National Energy Administration of China; the producers are not engaged in any bidding process.

Besides, the case in China is slightly different: ancillary services in China are categorized into “basic” ancillary services (PFC, basic peak regulation and basic reactive power control) that are mandatory and provided free of charge, and “paid” ancillary services (AGC, spinning and standing reserve, paid peak regulation, paid reactive power control and black start) that receive compensation payments. Although in China the PFC service overall belong to free “basic” ancillary services, now HPPs are rewarded or fined according to regulation performance.

(2) Determination of payment value
Regarding the payment, in Sweden, it is determined before the regulation; while in the USA and China, it is determined after the regulation based on indicators of regulation performance. The difference here reflects different payment philosophies.

Supplementary Note 8

- Contribution payment

The electrical energy that contributes to the PFC regulation is shown in Supplementary Figure 20, for demonstrating the core idea of the contribution payment.

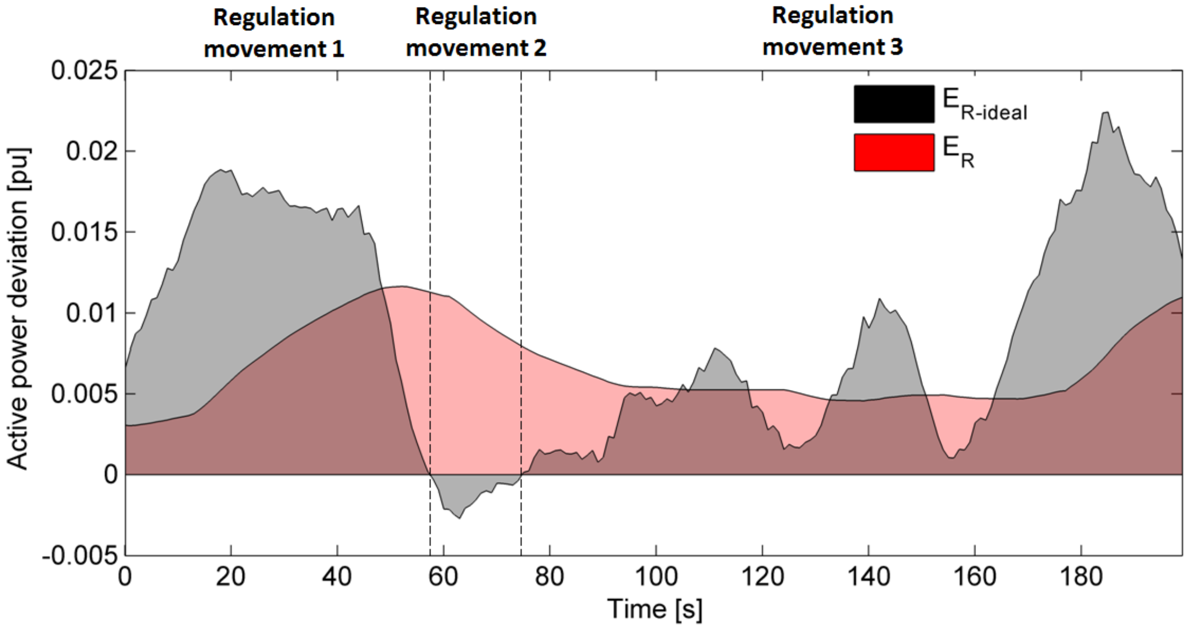

Supplementary Figure 20. Demonstration of the electrical energy that contributes to the PFC regulation: actual energy (in red) and ideal energy (in gray). The whole evaluation period (*i.e.* one day in this paper) is divided into many regulation movement periods. A regulation movement period is defined as the time spent between two neighbour zero-crossing points of frequency deviation. As an example, the score of regulation correctness of regulation movement 2 is 0, however it is not an effective regulation movement, hence its effect is ignored for calculating the contribution payment.

Effective regulation movement

Here we expound the purpose of introducing the effective regulation movement. If the value of $E_{R\text{-}ideal}$ of a regulation movement exceeds a threshold of 0.2 s, it is deemed as an effective regulation movement; otherwise the movement is ignored for calculating the contribution payment. As an example, the regulation movement 2 in Supplementary Figure 20 is not an effective regulation movement.

The reason is to exclude the influence of numerous small regulation movements acting on the tiny frequency deviations. For HPPs in which the frequency dead-zone of turbine governors is applied, the actuators of turbines respond to frequency changes only if the frequency deviation exceeds the dead-zone, hence the effective regulation movement can be excluded, *e.g.* in HPPs in the USA and China. However, for power grids where the frequency dead-zone is not applied, *e.g.* for the Nordic power grid, considering the effective regulation movement is practical and necessary.

Detailed results of the contribution payment

Supplementary Table 7. Detailed simulation results of the contribution payment, including the ratio of contributed regulating energy ($\lambda_{R\text{-}avg}$) and the score of regulation correctness (λ_C)

HPP	Para.	Operation point	$P_{contrib.}$			$\lambda_{R\text{-}avg}$			λ_C		
			S1	S2	S3	S1	S2	S3	S1	S2	S3
HPP 1	Ep1	1	0.950	0.787	0.453	0.758	0.635	0.266	0.821	0.651	0.770
		2	0.974	0.818	0.510	0.778	0.664	0.306	0.836	0.663	0.845
		3	1.007	0.845	0.537	0.810	0.692	0.331	0.845	0.663	0.854
		4	1.075	0.895	0.579	0.881	0.739	0.375	0.836	0.675	0.851
		5	1.060	0.905	0.556	0.871	0.750	0.363	0.818	0.675	0.803
		6	0.960	0.817	0.495	0.772	0.661	0.315	0.809	0.672	0.746
		7	0.973	0.816	0.531	0.780	0.660	0.329	0.830	0.672	0.839
		Average	1.000	0.841	0.523	0.807	0.686	0.326	0.828	0.667	0.815
	Ep2	1	0.644	0.545	0.359	0.471	0.387	0.185	0.728	0.662	0.717
		2	0.644	0.553	0.375	0.472	0.394	0.192	0.723	0.668	0.754
		3	0.659	0.569	0.390	0.490	0.411	0.211	0.717	0.662	0.739
		4	0.694	0.599	0.415	0.524	0.438	0.234	0.719	0.677	0.746
		5	0.696	0.609	0.412	0.525	0.448	0.236	0.726	0.679	0.726
		6	0.654	0.571	0.385	0.483	0.411	0.214	0.721	0.673	0.708
		7	0.651	0.570	0.389	0.482	0.410	0.211	0.712	0.670	0.735
		Average	0.663	0.574	0.389	0.492	0.414	0.212	0.721	0.670	0.732
	Ep3	1	0.264	0.177	0.224	0.108	0.027	0.064	0.640	0.611	0.652
		2	0.263	0.176	0.222	0.107	0.027	0.062	0.638	0.607	0.652
		3	0.264	0.179	0.224	0.108	0.028	0.065	0.640	0.613	0.652
		4	0.267	0.184	0.227	0.111	0.033	0.068	0.636	0.614	0.647
		5	0.271	0.191	0.234	0.115	0.040	0.072	0.638	0.616	0.659
		6	0.267	0.189	0.228	0.111	0.038	0.070	0.638	0.616	0.647
		7	0.260	0.184	0.226	0.106	0.032	0.066	0.631	0.616	0.650
		Average	0.265	0.183	0.226	0.110	0.032	0.067	0.637	0.613	0.651
HPP 2	Ep1	1	0.911	0.820	0.474	0.923	0.836	0.375	0.851	0.746	0.866
		2	0.997	0.917	0.574	1.040	0.964	0.533	0.809	0.716	0.731
		3	0.854	0.757	0.438	0.860	0.774	0.372	0.818	0.681	0.699
		4	0.881	0.793	0.470	0.889	0.809	0.382	0.839	0.719	0.818
		5	0.932	0.826	0.491	0.949	0.845	0.393	0.851	0.740	0.878
		6	0.774	0.703	0.280	0.781	0.707	0.212	0.734	0.681	0.546
		7	0.798	0.700	0.388	0.786	0.704	0.288	0.836	0.675	0.782
		Average	0.878	0.788	0.445	0.890	0.805	0.365	0.820	0.708	0.760
	Ep2	1	0.559	0.495	0.319	0.513	0.445	0.212	0.732	0.688	0.743
		2	0.598	0.544	0.377	0.566	0.505	0.290	0.721	0.690	0.717
		3	0.559	0.503	0.336	0.515	0.455	0.239	0.728	0.686	0.719
		4	0.549	0.492	0.331	0.502	0.442	0.230	0.728	0.686	0.732
		5	0.568	0.508	0.336	0.528	0.461	0.232	0.723	0.686	0.750
		6	0.538	0.483	0.287	0.492	0.433	0.190	0.715	0.675	0.673
		7	0.511	0.460	0.292	0.458	0.408	0.183	0.719	0.664	0.723
		Average	0.555	0.498	0.325	0.511	0.450	0.225	0.724	0.682	0.723
	Ep3	1	0.214	0.152	0.182	0.105	0.033	0.062	0.645	0.623	0.658
		2	0.214	0.157	0.183	0.106	0.039	0.069	0.643	0.627	0.636
		3	0.221	0.167	0.198	0.114	0.052	0.079	0.645	0.627	0.668
		4	0.206	0.153	0.191	0.096	0.036	0.073	0.643	0.618	0.658
		5	0.200	0.145	0.189	0.091	0.027	0.072	0.634	0.616	0.654
		6	0.204	0.150	0.190	0.092	0.031	0.076	0.645	0.625	0.643
		7	0.192	0.143	0.184	0.079	0.022	0.064	0.640	0.627	0.661
		Average	0.207	0.152	0.188	0.098	0.034	0.071	0.642	0.623	0.654

Supplementary Note 9

- Brief introduction for impact of the efficiency changes in regulation

It is worth emphasizing that even a minor efficiency loss is crucial in hydropower operations because of the significant installed capacity of individual units. Normally, a project will be initiated if it can improve the efficiency by 0.2 %[37], and 0.5 % efficiency loss from an original efficiency guarantee is regarded as significant. Examples are given as follows.

(1) For HPP 1 with 52.0 MW capacity, 0.5 % efficiency loss corresponds to 1.82 GWh per year if 80 % of its capacity is utilized.

(2) Shuikou HPP in China contains seven 200 MW Kaplan turbines and has generated more than 1000 TWh electricity in 20 years from 1993 to 2013; 0.5 % efficiency loss corresponds to 5 TWh energy loss.

(3) A preliminary estimation for the whole Nordic power system in this paper: the total hydropower installed capacity in the Nordic countries is 46 500 MW; the sum of regulating hydropower capacity varies depending on the demand and power market situation, etc; while an estimation of average amount of regulating hydropower is 37650 MW (the value of power base of the grid model)[27,28], 0.5 % efficiency loss leads to 188.25 MW power loss.

Supplementary Note 10

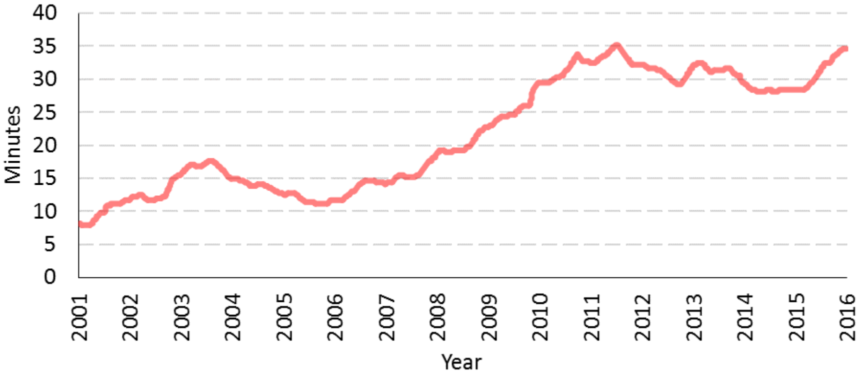

Supplementary Figure 21. Development in frequency quality of the Nordic power grid: the number of minutes per week (floating average of 52 weeks) with frequency outside 49.9 – 50.1 Hz from 2001 to the first quarter of 2016[38].

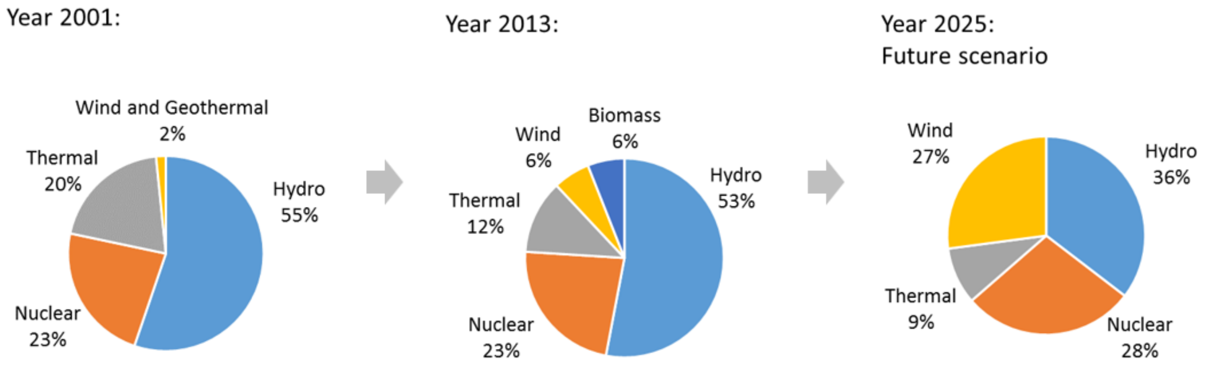

Supplementary Figure 22. For the Nordic power system: power generation in 2001[39] and 2013[40], and power capacity scenario for 2025[41]

Frequency sequences for future scenarios

Changes in the Nordic power system from now to 2025 will lead to an additional deterioration of frequency quality if appropriate measures are not adopted[38]. Hence, we firstly simulate grid frequency sequences for present and future scenarios, and then apply them to quantitatively investigate the influence of VRE sources. More exactly, as shown in Figure 2 in the main body of the paper, we adopt different frequency inputs for future scenarios as presented in Supplementary Table 8.

Supplementary Table 8. Detailed information of four scenarios: the inertia, the damping constant, the standard deviation of net load and the number of minutes per day for the frequency outside 49.9 – 50.1 Hz.

Scenario	System inertia, M [s]	Damping constant, D [pu]	Standard deviation of net load [pu]	Minutes for frequency outside 49.9-50.1 Hz
Scenario 1 (current)	14.6	0.66	0.010	52.4
Scenario 2	7.3	0.66	0.010	60.6
Scenario 3	14.6	0.33	0.010	70.4
Scenario 4	14.6	0.66	0.015	264.1
Scenario 5	7.3	0.33	0.015	314.2

First, we expound the Scenario 1 for the current condition. The frequency sequence of Scenario 1 is the original measured data shown in Figure 2, and the time for the frequency outside the bandwidth in the day is 52.4 minutes, which is larger than the corresponding value during the first three months in 2016 (35.7 minutes shown in Supplementary Figure 21).

Then for Scenarios 2 – 5, the frequency sequences are affected by VRE in terms of the three main factors: the inertia, the damping constant and the standard deviation of the net load. Representative and intermediate values of these three parameters are chosen for future scenarios.

(1) The inertia (Scenario 2): the decreased inertia indicates the reduced kinetic energy for future scenarios. As introduced in the main body of this paper, the Nordic TSOs estimate that the kinetic energy of the system during low loads will be 124 GWs in 2020 and possibly as low as 80 GWs in 2025[41], and the value for the current system is 250 GWs. Here we set the inertia to 50 % of the value for current case.

(2) The damping constant (Scenario 3): the decreased damping constant reflects the influence on electro-mechanical oscillations of the power system. As introduced in the main body of this paper, for influence from VRE penetration on electro-mechanical oscillations in power systems, both detrimental and beneficial impacts are possible[42,43]. Here we investigate the detrimental effect as an example by setting the damping constant to 50 % of the value for current case.

(3) The standard deviation of the net load (Scenario 4): the increased standard deviation of the net load represents for the enlarged VRE variability. As introduced in the main body of this paper, standard deviations of the net load of the Nordic power system in various VRE scenarios based on relatively long-term time scales (> daily) are quantified, and the value could be larger than 200 % of the current case[30]. However, the explicit relationship between the VRE integration and net load variability in the sub-hourly scale is still unclear for the short-term scale analyse. Here we increase the standard deviation of the net load to 150 % of the value for current case.

The frequency sequences of Scenarios 2 – 5 are simulated by applying Model 2 (Supplementary Figure 1) based on the system parameters, the detailed method is described in the part "Simulation of the frequency sequence" in Supplementary Note 6, and the scaling factor K_1 and K_2 are set to 0 and 0.4 respectively. It is shown that the corresponding time lengths for frequency outside the bandwidth are enlarged under Scenarios 2-5, the Scenario 5 has the most deteriorative frequency fluctuations, because all three factors are included in Scenario 5.

Detailed comparisons between the frequency sequences of Scenario 1 (original measured frequency) and Scenario 5 (simulated by Model 2) are shown in Supplementary Figure 23; it is clearly demonstrated that, under Scenario 5, the frequency deviation increases and the time of the grid frequency outside of the frequency band (49.9 Hz – 50.1 Hz) becomes longer.

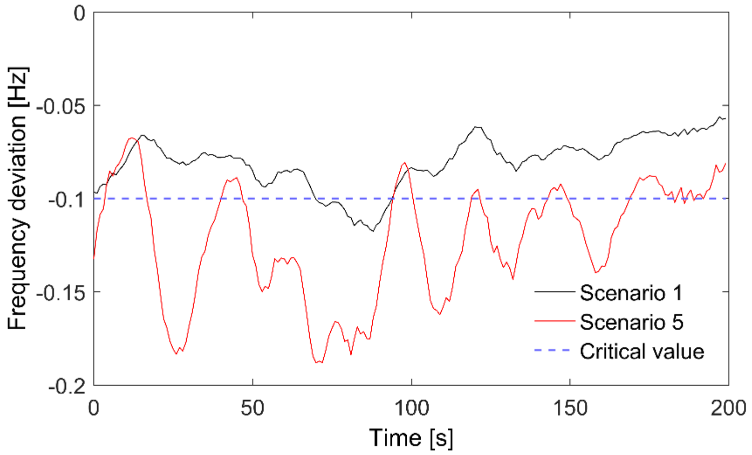

(a)

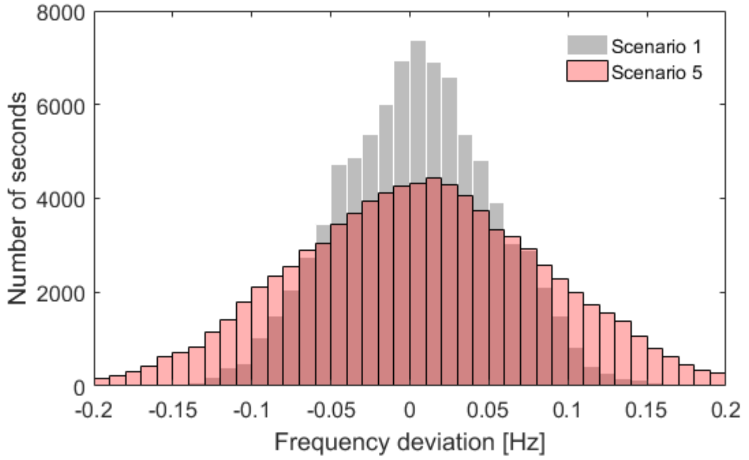

(b)

Supplementary Figure 23. Frequency deviation sequences of Scenario 1 and Scenario 5: (a) time domain demonstration for 200 seconds; (b) Histogram. ±0.1 Hz of the frequency deviation is the frequency bandwidth.

Supplementary Table 9. Detailed simulation results of burden under different scenarios

Burden	Strategy	Scenario	HPP 1			HPP 2		
			Ep1	Ep2	Ep3	Ep1	Ep2	Ep3
Distance -GV movement [/]	S1-S3	1	7.610	15.634	31.829	7.610	15.634	31.829
		2	9.179	18.609	38.158	9.179	18.609	38.158
		3	9.275	19.008	38.623	9.275	19.008	38.623
		4	11.376	23.158	46.858	11.376	23.158	46.858
		5	18.906	37.522	76.028	18.906	37.522	76.028
Amount - GV movement [/]	S1-S3	1	2583	2595	2750	2583	2595	2750
		2	4114	4109	4410	4114	4109	4410
		3	2659	2679	2793	2659	2679	2793
		4	2427	2586	2835	2427	2586	2835
		5	4271	4324	4592	4271	4324	4592
Distance - RB movement [/]	S1	1	4.489	11.436	25.925	6.791	16.128	34.751
		2	4.653	12.369	29.153	7.240	17.827	39.486
		3	5.497	14.016	31.732	8.278	19.730	42.172
		4	6.879	16.052	37.322	10.138	21.169	40.960
		5	10.423	25.120	58.872	15.760	32.869	64.394
	S2	1	0.443	1.824	7.041	0.878	3.486	11.605
		2	0.448	1.814	6.948	0.878	3.455	11.327
		3	0.493	2.006	8.176	0.966	3.841	13.719
		4	0.869	3.428	13.181	1.597	5.140	15.639
		5	0.956	3.845	15.902	1.739	5.818	19.018
	S3	All	0	0	0	0	0	0
Amount - RB movement [/]	S1	1	1141	1509	1777	1259	1579	1825
		2	1531	2347	2895	1867	2567	2971
		3	1437	1813	2087	1589	1919	2089
		4	1293	1649	2559	1399	1731	1971
		5	2767	3322	4068	3056	3456	3688
	S2	1	21	45	192	31	75	332
		2	21	45	172	31	77	314
		3	23	49	254	33	85	408
		4	29	73	610	43	125	391
		5	33	95	752	45	163	556
	S3	All	0	0	0	0	0	0
Efficiency change [pu]	S1	1	-0.079%	-0.217%	-0.726%	-0.030%	-0.125%	-0.528%
		2	-0.078%	-0.217%	-0.725%	-0.030%	-0.125%	-0.528%
		3	-0.082%	-0.228%	-0.770%	-0.033%	-0.133%	-0.562%
		4	-0.141%	-0.428%	-1.466%	-0.073%	-0.104%	-0.430%
		5	-0.148%	-0.453%	-1.536%	-0.078%	-0.107%	-0.457%
	S2	1	-0.097%	-0.249%	-0.771%	-0.061%	-0.153%	-0.555%
		2	-0.097%	-0.249%	-0.769%	-0.063%	-0.154%	-0.554%
		3	-0.101%	-0.260%	-0.817%	-0.062%	-0.159%	-0.588%
		4	-0.168%	-0.464%	-1.540%	-0.293%	-0.320%	-1.559%
		5	-0.174%	-0.488%	-1.608%	-0.313%	-0.340%	-1.646%
	S3	1	-0.144%	-0.535%	-1.941%	-0.196%	-0.743%	-2.505%
		2	-0.144%	-0.534%	-1.939%	-0.195%	-0.742%	-2.503%
		3	-0.153%	-0.569%	-2.048%	-0.209%	-0.788%	-2.628%
		4	-0.308%	-1.159%	-3.546%	-1.137%	-1.147%	-4.193%
		5	-0.327%	-1.229%	-3.668%	-1.205%	-1.207%	-4.335%

Values in the cells are average values for seven operating points. For S3, the distance and amount of RB are zero under all the operating conditions.

Supplementary Table 10. Detailed simulation results of the regulation payments under different scenarios

Payment	Strategy	Scenario	HPP 1			HPP 2		
			Ep1	Ep2	Ep3	Ep1	Ep2	Ep3
Strength [pu]	/	All	Does not change with different scenarios					
Mileage [pu]	S1	1	100.0%	221.5%	467.6%	89.6%	194.0%	396.1%
		2	112.8%	251.5%	544.3%	102.9%	224.0%	465.9%
		3	121.1%	269.6%	567.9%	109.1%	236.5%	480.8%
		4	156.1%	336.8%	672.4%	138.4%	289.4%	554.6%
		5	247.1%	531.0%	1067.5%	224.1%	460.9%	876.9%
	S2	1	55.3%	119.7%	267.3%	48.6%	105.1%	236.2%
		2	66.7%	139.1%	306.0%	58.3%	121.6%	269.2%
		3	65.8%	141.9%	317.9%	57.9%	124.7%	283.0%
		4	85.6%	186.7%	431.6%	75.4%	163.9%	366.2%
		5	134.5%	278.0%	636.9%	117.6%	244.9%	534.0%
	S3	1	49.7%	102.8%	207.3%	44.2%	94.8%	202.4%
		2	61.3%	123.7%	251.7%	54.5%	114.1%	245.8%
		3	60.0%	124.8%	249.9%	53.8%	115.6%	245.5%
		4	74.7%	153.6%	277.5%	68.0%	145.6%	267.3%
		5	124.8%	249.2%	448.4%	114.4%	238.7%	423.8%
Contribution [pu]	S1	1	100.0%	66.3%	26.5%	87.8%	55.5%	20.7%
		2	103.4%	72.6%	36.6%	91.3%	61.4%	30.0%
		3	106.7%	73.8%	34.2%	94.0%	62.1%	27.2%
		4	117.2%	76.9%	29.7%	126.5%	78.5%	28.1%
		5	126.8%	88.7%	45.6%	136.9%	91.5%	45.0%
	S2	1	84.1%	57.4%	18.3%	78.8%	49.8%	15.2%
		2	85.5%	60.0%	23.5%	80.2%	51.8%	20.0%
		3	88.5%	61.3%	21.8%	82.8%	53.6%	18.4%
		4	103.3%	66.8%	18.3%	114.1%	70.8%	18.6%
		5	108.9%	72.6%	27.4%	119.0%	76.8%	27.8%
	S3	1	52.3%	38.9%	22.6%	44.5%	32.5%	18.8%
		2	53.6%	41.4%	26.6%	45.7%	34.8%	22.4%
		3	55.4%	42.6%	26.2%	47.3%	35.7%	21.9%
		4	57.5%	41.5%	22.6%	61.5%	43.0%	23.0%
		5	60.9%	45.7%	28.8%	65.3%	48.0%	30.0%

Values in the cells are average values for seven operating points. The value of the strength payment is not given because it does not change with different scenarios.

Supplementary References

1. Demello, F. *et al.* Hydraulic-turbine and turbine control-models for system dynamic studies. *IEEE Trans. Power Syst.* **7**, 167-179 (1992).
2. De Jaeger, E., Janssens, N., Malfliet, B. & Van De Meulebroeke, F. Hydro turbine model for system dynamic studies. *IEEE Trans. Power Syst.* **9**, 1709-1715 (1994).
3. Strah, B., Kuljaca, O. & Vukic, Z. Speed and active power control of hydro turbine unit. *IEEE Trans. Energ. Convers.* **20**, 424-434 (2005).
4. Pennacchi, P., Chatterton, S. & Vania, A. Modeling of the dynamic response of a Francis turbine. *Mech. Syst. Signal Process.* **29**, 107-119 (2012).
5. Chen, D. *et al.* Nonlinear dynamic analysis for a Francis hydro-turbine governing system and its control. *J. Franklin Inst.* **351**, 4596-4618 (2014).
6. Giosio, D.R., Henderson, A.D., Walker, J.M. & Brandner, P.A. Physics Based Hydraulic Turbine Model for System Dynamics Studies. (2016).
7. Souza Jr, O., Barbieri, N. & Santos, A. Study of hydraulic transients in hydropower plants through simulation of nonlinear model of penstock and hydraulic turbine model. *IEEE Trans. Power Syst.* **14**, 1269-1272 (1999).
8. Nicolet, C. *et al.* High-order modeling of hydraulic power plant in islanded power network. *IEEE Trans. Power Syst.* **22**, 1870-1880 (2007).
9. Fang, H., Chen, L., Dlakavu, N. & Shen, Z. Basic modeling and simulation tool for analysis of hydraulic transients in hydroelectric power plants. *IEEE Trans. Energ. Convers.* **23**, 834-841 (2008).
10. Zeng, Y., Guo, Y., Zhang, L., Xu, T. & Dong, H. Nonlinear hydro turbine model having a surge tank. *Math. Comput. Model. of Dynam. Syst.* **19**, 12-28 (2013).
11. Yang, W. *et al.* A Mathematical Model and Its Application for Hydro Power Units under Different Operating Conditions. *Energies* **8**, 10260-10275 (2015).
12. Guo, W., Yang, J., Wang, M. & Lai, X. Nonlinear modeling and stability analysis of hydro-turbine governing system with sloping ceiling tailrace tunnel under load disturbance. *Energ. Conv. Manage.* **106**, 127-138 (2015).
13. Mansoor, S., Jones, D., Bradley, D.A., Aris, F. & Jones, G. Reproducing oscillatory behaviour of a hydroelectric power station by computer simulation. *Contr. Eng. Pract.* **8**, 1261-1272 (2000).
14. Pérez-Díaz, J.I., Sarasúa, J.I. & Wilhelmi, J.R. Contribution of a hydraulic short-circuit pumped-storage power plant to the load–frequency regulation of an isolated power system. *Int. J. Elec. Power Energ. Syst.* **62**, 199-211 (2014).
15. Kishor, N., Saini, R. & Singh, S. A review on hydropower plant models and control. *Renew. Sust. Energ. Rev.* **11**, 776-796 (2007).
16. Brezovec, M., Kuzle, I. & Tomisa, T. Nonlinear digital simulation model of hydroelectric power unit with Kaplan turbine. *IEEE Trans. Energ. Convers.* **21**, 235-241 (2006).
17. Kranjcic, D. & Štumberger, G. Differential evolution-based identification of the nonlinear kaplan turbine model. *IEEE Trans. Energ. Convers.* **29**, 178-187 (2014).
18. Djukanovic, M. *et al.* Neural-net based coordinated stabilizing control for the exciter and governor loops of low head hydropower plants. *IEEE Trans. Energ. Convers.* **10**, 760-767 (1995).
19. Dobrijevic, D.M. & Jankovic, M.V. An approach to the damping of local modes of oscillations resulting from large hydraulic transients. *IEEE Trans. Energ. Convers.* **14**, 754-759 (1999).
20. Kosterev, D. Hydro turbine-governor model validation in pacific northwest. *IEEE Trans. Power Syst.* **19**, 1144-1149 (2004).
21. Zhao, J. *et al.* Dynamic Model of Kaplan Turbine Regulating System Suitable for Power System Analysis. *Math. Probl. Eng.* **2015**(2015).
22. Kundur, P., Balu, N.J. & Lauby, M.G. *Power system stability and control*, (McGraw-hill New York, 1994).
23. Yang, W. *et al.* Wear and tear on hydro power turbines–Influence from primary frequency control. *Renew. Energ.* **87**, 88-95 (2016).

24. Yang, W. *et al.* Wear reduction for hydropower turbines considering frequency quality of power systems: A study on controller filters. *IEEE Trans. Power Syst.* **32**, 1191-1201 (2017).
25. Ghrist III, W.D. Floating deadband for speed feedback in turbine load control. (ed. USPTO) (1986).
26. MATLAB Documentation. http://se.mathworks.com/help/simulink/slref/backlash.html.
27. Saarinen, L. Uppsala University (2014).
28. Saarinen, L., Norrlund, P. & Lundin, U. Tuning primary frequency controllers using robust control theory in a power system dominated by hydropower. in *CIGRE Session 2016* (Paris, France, 2016).
29. Söder, L. Simplified analysis of balancing challenges in sustainable and smart energy systems with 100% renewable power supply. *WIREs Energ. Environ.* **5**, 401-412 (2016).
30. Olauson, J. *et al.* Net load variability in Nordic countries with a highly or fully renewable power system. *Nat. Energ.* **1**, 16175 (2016).
31. IEC 60193:1999 Hydraulic turbines, storage pumps and pump-turbines - Model acceptance tests. (1999).
32. Kercan, V., Djelic, V., Rus, T. & Vujanic, V. Experience with Kaplan turbine efficiency measurements–Current meters and/or index test flow measurement. in *Proc. IGHEM* (Montreal, Canada, 1996).
33. Adamkowski, A., Lewandowski, M. & Lewandowski, S. Selected Experiences with Optimization Tests of the Kaplan-Type Hydraulic Turbines. *J. Energ. Power Eng.* **8**(2014).
34. Ljung, L. & Glad, T. *Control theory-multivariable and nonlinear methods*, (Taylor and Francis, 2000).
35. SvK document 2015/1057, Regler för upphandling och rapportering av FCR-N och FCR-D (in Swedish). (2015).
36. PJM Manual 11: Energy & Ancillary Services Market Operations, Revision: 89. (Forward Market Operations, 2017).
37. Hydro Life Extension ModernizationGuides - Volume 2: Hydromechanical Equipment. (EPRI, 2000).
38. Challenges and Opportunities for the Nordic Power System. (Svenska kraftnät, Statnett, Fingrid and Energinet.dk, 2016).
39. Holttinen, H. Helsinki University of Technology (2003).
40. NordREG. Nordic Market Report 2014 - Development in the Nordic Electricity Market http://www.nordicenergyregulators.org/wp-content/uploads/2014/06/Nordic-Market-Report-2014.pdf. (2014).
41. Ørum, E. *et al.* Future system inertia. (ENTSO- E, 2015).
42. Eftekharnejad, S., Vittal, V., Heydt, G.T., Keel, B. & Loehr, J. Impact of increased penetration of photovoltaic generation on power systems. *IEEE Trans. Power Syst.* **28**, 893-901 (2013).
43. Domínguez-García, J.L., Gomis-Bellmunt, O., Bianchi, F.D. & Sumper, A. Power oscillation damping supported by wind power: A review. *Renew. Sust. Energ. Rev.* **16**, 4994-5006 (2012).
